# A multisite super-crosslinked sulfur-heterocyclic polymer cathode for high-voltage and low-temperature aluminum–organic batteries

**DOI:** 10.1093/nsr/nwaf526

**Published:** 2025-11-22

**Authors:** Yuxi Guo, Ke Guo, Wei Wang, Zheng Huang, Yaxue Wang, Mingyong Wang, Yanli Zhu, Shuqiang Jiao

**Affiliations:** State Key Laboratory of Advanced Metallurgy, University of Science and Technology Beijing, Beijing 100083, China; State Key Laboratory of Explosion Science and Safety Protection, Beijing Institute of Technology, Beijing 100081, China; State Key Laboratory of Explosion Science and Safety Protection, Beijing Institute of Technology, Beijing 100081, China; State Key Laboratory of Advanced Metallurgy, University of Science and Technology Beijing, Beijing 100083, China; State Key Laboratory of Advanced Metallurgy, University of Science and Technology Beijing, Beijing 100083, China; State Key Laboratory of Advanced Metallurgy, University of Science and Technology Beijing, Beijing 100083, China; State Key Laboratory of Advanced Metallurgy, University of Science and Technology Beijing, Beijing 100083, China; State Key Laboratory of Explosion Science and Safety Protection, Beijing Institute of Technology, Beijing 100081, China; State Key Laboratory of Advanced Metallurgy, University of Science and Technology Beijing, Beijing 100083, China

**Keywords:** aluminum–ion batteries, organic cathode materials, molecular tailoring, high operating voltage, low-temperature durability

## Abstract

Simultaneously attaining high energy density and long cycling life remains a critical challenge for aluminum–organic batteries (AOBs) due to low operating voltage, limited active sites and unstable coordination structure of organic cathodes. Herein, we design a multisite super-crosslinked sulfur-heterocyclic polymer cathode. The electronegative sulfur heterocycles can significantly weaken the electron-donating effect, promoting the operating voltage to 2.0 V (average ∼1.7 V), which is a breakthrough for AOBs (<1.5 V for almost all AOBs). Tailoring the linking patterns of polymers to increase active sites can maximize redox activity to 12-electron-transfer, contributing to a high capacity of 150 mAh g^−1^. The designed organic cathode achieves 255 Wh kg^−1^ energy density, breaking the upper limit of conventional graphite cathodes (∼200 Wh kg^−1^). Notably, the weak coordination interaction between C‒S^+^‒C radicals and AlCl_4_^−^ carriers ensures structural stability, enabling the battery’s excellent low-temperature durability, with almost 100% capacity retention after 12 000 cycles at −20°C.

## Teaser

This work designs a multisite, super-crosslinked sulfur-heterocyclic polymer cathode with weak electron-donating effect and multi-electron transfer capability, thus achieving high operating voltage and superior low-temperature durability in aluminum–organic batteries.

## INTRODUCTION

Rechargeable aluminum-ion batteries (AIBs) are highly desirable devices for large-scale energy storage, owing to the abundant reserves, low cost, high theoretical capacity and fewer dendrites of Al metal [1,2]. Unlike other alkali metal-ion batteries, due to the high charge density and strong complexation of trivalent Al^3+^ ions, AIBs generally use large-sized Al-complex anions (such as AlCl_4_^−^ and Al_2_Cl_7_^−^) as charge carriers in ionic liquid electrolytes [3,4]. This presents a serious challenge for developing suitable cathode materials. Graphitic cathode materials show broad application prospects because of their high operating voltages and good cycling stability [3,5]. However, the limited intercalation of AlCl_4_^−^ anions in graphite layers results in a low specific capacity (<120 mAh g^−1^). Although a variety of high-capacity cathode materials have been developed, such as chalcogens [6–8], transition metal chalcogenides [9–11] and MXene [12] etc., these inorganic materials based on a conversion reaction mechanism usually suffer from either low operating voltage, poor cycling stability or sluggish reaction kinetics. These issues hinder the development of inorganic cathode materials, prompting developers of AIBs to turn to organic cathode materials with more potential.

Recently, organic materials have attracted extensive attention in terms of electrochemical energy storage due to their diverse redox activity, flexible structure designability and unique charge storage mechanism [13,14]. In contrast to inorganic materials, organic materials can store charge carriers on their redox-active motifs through coordination reactions, which is generally unaffected by the type and size of carriers, showing great potential for storing large-sized Al-complex ions. According to the stored charge species, organic electrode materials can be categorized into n-type (coordinated with cations), p-type (coordinated with anions) and bipolar-type (coordinated with cations and anions alternately) [14]. In the redox process of organic materials, electron gain and loss occur in the lowest unoccupied molecular orbital (LUMO) and the highest occupied molecular orbital (HOMO), which determines the redox potential [15]. N-type organic materials generally accept electrons in the LUMO orbital for reduction reactions to store cations. Carbonyl compounds are the most extensively studied n-type organic cathodes in AIBs, such as quinones [16, 17], imides [18] and ketones [19], which can produce anionic radicals (C–O^−^) via the enolization of carbonyl (C=O) groups to coordinate with Al-complex cations (AlCl_2_^+^ or AlCl^2+^). Besides this, some nitrogen-containing compounds including imines (C=N) [20,21], nitriles (C≡N) [22] and azo compounds (N=N) [23] can also coordinate with AlCl_2_^+^ cations. These n-type organic materials offer high specific capacity, but suffer from low discharge voltages and poor cycling stability due to unstable coordination structures caused by repeated bond rearrangements. In contrast to n-type organic materials, p-type organic materials typically lose electrons in the lower-level HOMO for oxidation reactions to store anions, theoretically offering higher discharge voltages [24–26]. For example, polypyrenes can reversibly store AlCl_4_^−^ anions through charge delocalization within their molecular skeletons, delivering a high discharge voltage of ∼1.7 V [24]. However, they exhibit a low specific capacity due to limited active sites and single-electron redox reaction. In addition, bipolar-type organic materials have the characteristics of both n-type and p-type organic materials, involving the alternate storage of dual ions (AlCl_4_^−^ and AlCl_2_^+^) at different active sites, exhibiting medium discharge voltages and specific capacities [27,28]. Nevertheless, to date, most organic cathode materials in AIBs fail to deliver a high operating voltage (>1.5 V) have a high specific capacity (>150 mAh g^−1^), which limits their energy density. This must be improved through ingenious molecular design. Furthermore, the severe dissolution of small organic molecules in acidic ionic liquid electrolytes also leads to rapid capacity decay, thus hindering their practical application [16,19]. Therefore, it remains the most critical challenge to develop organic cathode materials with high energy density and long cycling life.

Herein, we design a multisite super-crosslinked sulfur-heterocyclic polymer cathode with 12-electron-transfer for high-energy and long-life aluminum–organic batteries (AOBs). The high-electronegativity sulfur-heterocyclic active units significantly reduce the electron-donating effect, promoting the redox potential. Furthermore, by tailoring the linking patterns, the enhanced and electronic/ionic conductivity can maximize redox activity, thereby increasing the specific capacity. As a result, the optimized polythianthrene-4 (PTT-4) cathode delivers a high voltage of 2.0 V (average ∼1.7 V), and a high capacity of 150 mAh g^−1^, corresponding to an energy density of 255 Wh kg^−1^. Impressively, benefiting from the weak coordination interaction between C‒S^+^‒C cationic radicals and AlCl_4_^−^ anionic carriers, which guarantees high structural stability and fast reaction kinetics. The constructed Al||PTT-4 battery demonstrates excellent cycling stability at a low temperature of −20°C, with a capacity retention of ∼100%, even after 12 000 cycles. Both experimental characterizations and theoretical calculations confirm a two-step 12-electron-transfer reaction mechanism with dual-electron redox for each active unit. Our findings provide a new insight for designing a rational molecular structure to realize high-energy AOBs with low temperature durability.

## RESULTS AND DISCUSSION

### Molecular design for high-energy organic cathodes

**Figure 1.**
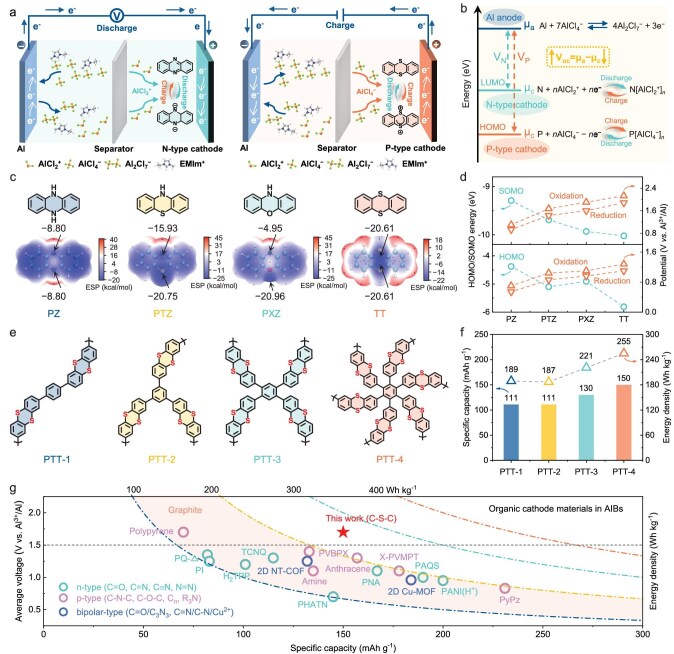
Molecular design for high-energy organic cathodes. (a) Charge storage mechanism of n-type and p-type organic cathodes in AIBs. (b) Scheme of the relationship between open-circuit voltage (V_oc_) for AIBs and organic cathode potential (µ_c_): V_oc_ = µ_a_ − µ_c_, which depends on the LUMO/HOMO energy levels of n-type/p-type organic cathodes. (c) Molecular structures and simulated ESP distributions of PZ, PTZ, PXZ and TT. The cyan and orange balls represent the minimum value and maximum value points, respectively. (d) Relationship of calculated HOMO/SOMO energies and tested first/second redox potentials. (e) Molecular structures of PTT-*n* (*n* = 1, 2, 3, 4). (f) Comparison of specific capacity and energy density. (g) Comparison of average voltage, specific capacity and energy density of the PTT-4 cathode with reported organic and graphite cathode materials in AIBs.

High operating voltage together with high capacity is highly desirable for batteries to obtain high energy density. On the one hand, the redox potential of organic materials is determined by the ability to gain or lose electrons, which depends on their frontier molecular orbital energy levels [13,15]. In AIBs, n-type organic cathodes typically undergo reduction reactions by accepting electrons in the LUMO level and coordinating with Al-complex cations (such as AlCl_2_^+^), while p-type organic cathodes undergo oxidation reactions by losing electrons from the lower HOMO level and coordinating with Al-complex anions (such as AlCl_4_^−^) (Fig. 1a and b). By decreasing the LUMO or HOMO energy levels, a higher output voltage can be obtained theoretically (Fig. 1b). On the other hand, the specific capacity depends on the electron transfer number per molecular weight [13]. Increasing active-site density to increase electron transfer number via rational molecular structure design can obtain a high capacity. Therefore, developing low-orbital-energy organic cathodes with high active-site density is expected to achieve high-energy AOBs.

To produce a high-voltage organic cathode, we use heteroatom substitution to reduce the HOMO level of p-type organic molecules. A series of conjugated heterocyclic molecules, including dihydrophenazine (PZ), phenothiazine (PTZ), phenoxazine (PXZ) and thianthrene (TT), are selected as the cathode materials for AIBs (Fig. 1c). The molecular electrostatic potential (ESP) is calculated to predict their active sites. It can be seen that the heterocyclic regions show obvious negative ESP values, which can act as the active sites for storing charge carriers. As the N heteroatom is replaced by S or O heteroatoms, the ESP values in PTZ (−20.75 kcal mol^−1^) and PXZ (−20.89 kcal mol^−1^) are much more negative than that of PZ (−8.80 kcal mol^−1^), indicating significantly enhanced electronegativity. Notably, TT (−20.61 kcal mol^−1^) with two S heteroatoms exhibits the highest electronegativity. The increase in electronegativity makes it more difficult for TT to lose electrons from the HOMO, thus leading to higher redox potentials. As the p-type redox reaction loses electrons from the highest energy level, the first redox potential is determined by the HOMO level, and the second is related to the singly occupied molecular orbital (SOMO) (Fig. S1a) [29]. Cyclic voltammetry (CV) curves show that each molecule displays two redox potential couples (Fig. S1b). Figure 1d reveals the relationship between the redox potentials and HOMO/SOMO energy levels of heterocyclic molecules. With the substitution of S or O heteroatoms, the HOMO/SOMO energy levels decrease, while the oxidation/reduction potentials increase. Among all molecules, TT exhibits the lowest HOMO (−5.81 eV) and SOMO (−10.02 eV) energy levels, indicating a weak electron-donating effect, which can offer the highest redox potentials [29,30]. Furthermore, the galvanostatic discharge curves also show two clear discharge voltage plateaus for each molecule (Fig. S1c). Among them, TT exhibits the highest operating voltage (1.82/1.08 V) and specific capacity (123 mAh g^−1^). The corresponding dQ/dV plots show a similar potential trend for PZ < PTZ < PXZ < TT (Fig. S1d), which is consistent with the CV results. Therefore, the TT molecule is selected as the active unit to design high-energy organic cathode materials.

To further achieve high specific capacity, we demonstrate a tailoring strategy for the linking patterns of sulfur-heterocyclic polymers. As shown in Fig. 1e, four polymers with different linkers are designed and synthesized, which are denoted as PTT-*n* (*n* = 1, 2, 3, 4). By increasing the linking number of active units from PTT-1 to PTT-4, the polymer structure transforms from a dimer to a hexamer. The increased density of the active site maximizes the electron transfer number from 4 to 12 electrons, contributing a high specific capacity and energy density (Fig. 1f). Furthermore, the regulation of linking patterns changes the electronic and spatial structure of the polymers, improves the electronic and ionic conductivity, and thus improves utilization rate of active sites (Table S1). Benefiting from the high operating voltage (1.7 V) and specific capacity (150 mAh g^−1^), the designed PTT-4 cathode achieves a high energy density of up to 255 Wh kg^−1^, which is superior to most reported organic cathode materials in AIBs and breaks through the limitation of conventional graphite cathodes (100−200 Wh kg^−1^) (Fig. 1g and Table S2).

The PTT-*n* (*n* = 1, 2, 3, 4) polymers are synthesized via Suzuki coupling reaction of TT active units and benzene linkers (Fig. S2). The ^1^H and ^13^C nuclear magnetic resonance (NMR) spectra verify the successful synthesis of two intermediates of the TT monomer (Figs S3 and S4). The molecular structure and chemical composition of the polymers are investigated using a variety of characterization methods. Fourier transform infrared (FTIR) spectra and solid-state ^13^C NMR are used to identify the molecular structure. In the FTIR spectra (Fig. S5), the characteristic absorption peaks of TT in the range from 600 to 1600 cm^−1^ are well retained for the polymers. The peak at about 870 cm^−1^ is assigned to C‒S‒C groups of TT [31,32]. Notably, the new peaks at 1685 cm^−1^ can be ascribed to the bending vibration of C=C groups of benzene linkers [33], indicating the successful coupling of TT units with benzenes. In the ^13^C NMR spectra (Fig. S6), the resonance signals at 128 and 135.5 ppm correspond to the C atoms of benzenes [33], and the signals at about 139 ppm belong to the C atoms of C‒S‒C groups. Furthermore, the X-ray photoelectron spectroscopy (XPS) spectra and elemental analysis show the presence of C and S elements (Fig. S7 and Table S3). These results confirm the successful synthesis of PTT-*n* (*n* = 1, 2, 3, 4) polymers.

Scanning electron microscopy (SEM) is used to characterize the morphology of the TT monomer and PTT-*n* (*n* = 1, 2, 3, 4) polymers (Figs S8 and S9). Compared with large-sized TT micron blocks, the prepared polymers show smaller nanosheet morphology, which can facilitate the effective utilization of active sites. X-ray diffraction (XRD) patterns show the obvious crystallinity characteristics of the TT monomer and the amorphous characteristics of the polymers (Fig. S10). Compared with the tightly packed small molecules, the amorphous flexible polymer structure is more conducive to the diffusion of active ions [24]. N_2_ adsorption–desorption measurements indicate that all polymers show nanoscale micropore structure (Fig. S11). In addition, thermogravimetric analysis (TGA) curves show that the polymers have high thermal stability with an onset decomposition temperature over 400°C, which is much higher than that of TT (Fig. S12). These potential advantages, including flexibility, porosity and thermal stability, make PTT-*n* (*n* = 1, 2, 3, 4) polymers highly desirable cathode materials for AOBs.

### Electrochemical performance of Al||PTT-*n* (*n* = 1, 2, 3, 4) batteries

**Figure 2.**
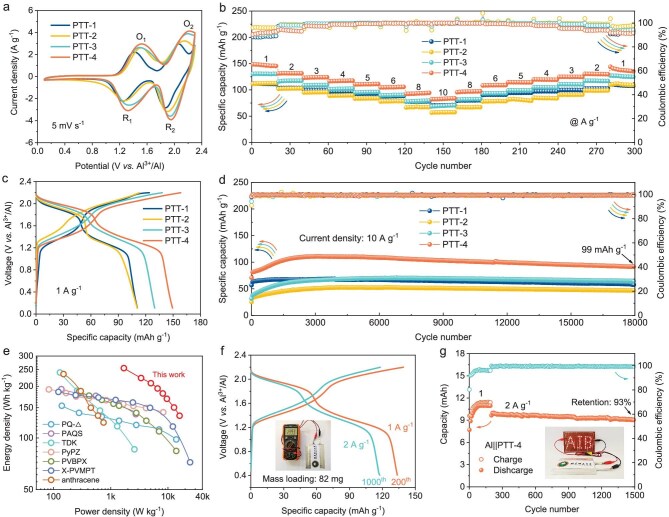
Electrochemical performance of Al||PTT-*n* (*n* = 1, 2, 3, 4) batteries. (a) CV curves at 5 mV s^−1^. (b) Rate performance at various current densities from 1 to 10 A g^−1^. (c) Galvanostatic discharge/charge curves at 1 A g^−1^. (d) Cycling performance at 10 A g^−1^. (e) Comparison of energy and power density of the PTT-4 cathode with recently reported organic cathodes in AIBs. (f) Galvanostatic discharge/charge curves and (g) cycling performance of the pouch-type Al||PTT-4 battery at 1 and 2 A g^−1^. The photos show the open-circuit voltage and continuous power supply capability of the pouch battery after being fully charged.

To evaluate the electrochemical performance of sulfur-heterocyclic polymers, the Al||PTT-*n* (*n* = 1, 2, 3, 4) batteries are assembled with PTT-*n* (*n* = 1, 2, 3, 4) as the cathode, Al metal as the anode, and AlCl_3_/[EMIm]Cl ionic liquid as the electrolyte. For comparison, an Al||TT battery with TT as the cathode is also assembled. CV curves are recorded to analyze the redox behavior of PTT-*n* (*n* = 1, 2, 3, 4) (Fig. 2a). All polymer cathodes display two reversible pairs of redox peaks in the voltage ranges of 1.8–2.2 V and 1.2–1.6 V, indicating a two-step redox reaction. Impressively, galvanostatic charge/discharge curves also show two distinct charge/discharge voltage plateaus at 1.8–2.2 V and 1.2–1.6 V for all polymer cathodes, with a high average operating voltage of ∼1.7 V (Fig. 2b). Such high output voltage mainly benefits from the low HOMO/SOMO energy levels and weak electron-donating effect of sulfur-heterocyclic molecules. Notably, the PTT-4 cathode delivers the highest specific capacity of 150 mAh g^−1^ at 1 A g^−1^, which is higher than that of PTT-1 (111 mAh g^−1^), PTT-2 (111 mAh g^−1^) and PTT-3 (130 mAh g^−1^) cathodes. The high capacity is attributed to the increased active-site density along with enhanced redox activity. Therefore, the optimized PTT-4 cathode achieves a high energy density of 255 Wh kg^−1^. In addition, the PTT-4 cathode shows good rate performance with capacities of 149, 132, 124, 117, 111, 105, 94 and 84 mAh g^−1^ at 1, 2, 3, 4, 5, 6, 8 and 10 A g^−1^, respectively, which is better than the other polymer cathodes (Fig. 2c and Fig. S13). The superior rate performance of PTT-4 can be attributed to its highly cross-linked spatial structure and intrinsic porous structure, which allows for fast transport of electrons and ions. Moreover, when the current density is returned to 1 A g^−1^, the capacity recovers to 144 mAh g^−1^, revealing the reversibility of the PTT-4 cathode.

Remarkably, all polymer cathodes exhibit a long cycling life over 18 000 cycles at 10 A g^−1^, showing higher cycling stability than the TT cathode (Fig. 2d and Fig. S14). In comparison, the PTT-4 cathode demonstrates a high specific capacity of 99 mAh g^−1^ after 18 000 cycles. The increase in capacity at the beginning of cycling can be attributed to the activation process of porous polymer materials [34]. Such long-term cycling stability benefits from the extended π-conjugated polymer structure, which inhibits the dissolution of TT molecules in the electrolyte (Figs S15 and S16) [26,35]. As a result, compared with recently reported organic cathode materials in AIBs, the PTT-4 cathode displays a considerably high energy density, along with a high power density (Fig. 2e and Table S2). Furthermore, the assembled pouch-type Al||PTT-4 battery delivers a high capacity of 11.0 and 9.7 mAh at 1 and 2 A g^−1^, respectively, and exhibits outstanding cycling stability, with a capacity retention of 93% after 1500 cycles (Fig. 2f and g and Fig. S17). The fully charged battery displays a stable open-circuit voltage of ∼2.0 V, which can power dozens of light-emitting diode (LED) lights. The battery can also operate stably at different bending states, showing good flexibility (Fig. S18). Additionally, the designed PTT-4 cathode also delivers a high energy density of 236 Wh kg^−1^ in AlCl_3_/urea electrolyte, as well as good cycling stability and rate capability (Fig. S19), indicating the universality of molecular design in energy storage. Considering the low cost and high sustainability of raw materials, such high-energy and long-life AOBs show promising application prospects in large-scale energy storage.

### Reaction kinetics and low-temperature performance

**Figure 3.**
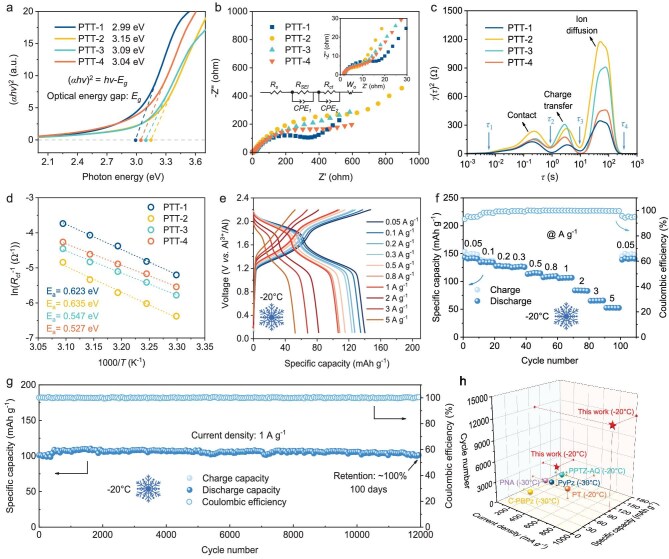
Reaction kinetics and low-temperature performance. (a) Calculated optical energy gaps (*E_g_*) by UV–vis spectra of PTT-*n* (*n* = 1, 2, 3, 4). (b) Nyquist plots of EIS and (c) corresponding DRT plots of PTT-*n* (*n* = 1, 2, 3, 4) cathodes in the fifth cycle. (d) Arrhenius plots of ln(Rct^−1^) vs. 1000/*T*. (e and f) Rate performance of PTT-4 cathode at various current densities from 0.05 to 5 A g^−1^ at −20°C. (g) Long cycling stability of PTT-4 cathode at 1 A g^−1^ at −20°C. (h) Comparison of low-temperature durability between the PTT-4 cathode and reported organic cathodes in AIBs.

To reveal the intrinsic electronic conductivity of PTT-*n* (*n* = 1, 2, 3, 4) polymers, ultraviolet–visible (UV–vis) spectroscopy is used to identify their optical energy gap (*E_g_*) (Fig. 3a and Fig. S20). The calculated *E_g_* values of PTT-1, PTT-2, PTT-3 and PTT-4 are 2.99, 3.15, 3.09 and 3.04 eV, respectively. With a lower *E_g_*, PTT-1 and PTT-4 have higher electronic conductivity, which contributes to faster redox reaction kinetics [36]. To further reveal the electrochemical reaction kinetics of PTT-*n* (*n* = 1, 2, 3, 4) cathodes, electrochemical impedance spectroscopy (EIS) is performed to investigate their charge transfer and ion diffusion behaviors. The Nyquist plot of all cathodes consists of two semicircles (ion migration resistance in solid electrolyte interface film *R_SEI_* and charge transfer resistance *R_ct_*) in the high-frequency region and a sloping line (ion diffusion resistance, *W_o_*) in the low-frequency region (Fig. 3b). The corresponding distribution of relaxation times (DRT) analysis suggests that both charge transfer and ion diffusion resistances show a tendency of first increasing and then decreasing from PTT-1 to PTT-4, which is consistent with the variation trend of *E_g_* calculated by UV–vis spectra (Fig. 3c). Notably, this impedance trend is attributed to competing effects: an initial increase due to greater steric hindrance in cross-linked polymers, followed by a decrease for PTT-4 due to its porous network structure and high active-site density, which enhance charge and ion transport [15]. Furthermore, with the increase of cycle number from 1 to 100 cycles, the fitting *R_SEI_* and *R_ct_* values of PTT-*n* (*n* = 1, 2, 3, 4) cathodes gradually decrease, indicating the continuously improved reaction kinetics (Figs S21–S23 and Table S4), which may be related to the activation process of polymers. Notably, the PTT-4 cathode shows the smallest charge transfer resistance after 100 cycles, exhibiting faster reaction kinetics than other polymer cathodes. Moreover, the activation energy (*E_a_*) for the charge transfer process of PTT-*n* (*n* = 1, 2, 3, 4) cathodes is also estimated by EIS at different temperatures (Fig. 3d). Based on the Arrhenius equation, the calculated *E_a_* values of PTT-1, PTT-2, PTT-3 and PTT-4 are 0.623, 0.635, 0.547 and 0.527 eV, respectively. The low *E_a_* value of PTT-4 ensures fast charge transfer and efficient utilization of the active sites. In addition, the capacity contributions of capacitive and faradaic processes of PTT-4 are analyzed by CV. The calculated *b* values are close to 1, indicating a predominantly capacitive charge storage behavior, which suggests fast reaction kinetics (Fig. S24). These results imply that the regulation of linking patterns changes the electronic and spatial structure of the polymers, improves the electronic and ionic conductivity, and thus accelerates the reaction kinetics.

The high-kinetics redox activity of the PTT-4 cathode encourages us to further explore its electrochemical performance at low temperature. Specifically, the PTT-4 cathode also shows good rate performance at −20°C, with capacities of 140, 135, 128, 124, 115, 109, 107, 82, 68 and 53 mAh g^−1^ at 0.05, 0.1, 0.2, 0.3, 0.5, 0.8, 1, 2, 3 and 5 A g^−1^, respectively (Fig. 3e). Moreover, when the current density is returned to 0.05 A g^−1^, the capacity recovers to 141 mAh g^−1^, showing good reversibility (Fig. 3f). The cycling performance of the PTT-4 cathode at −20°C is also tested after full activation. Impressively, the PTT-4 cathode exhibits extraordinary cycling stability with a capacity retention of ∼100%, even after 12 000 cycles (100 days) at 1 A g^−1^ (Fig. 3g). Furthermore, the PTT-4 cathode still maintains ∼87% capacity after 3000 cycles (158 days) at 0.2 A g^−1^ (Fig. S25). Compared with the reported organic cathode materials in AIBs, PTT-4 shows prominent low-temperature durability (Fig. 3h), which is mainly attributed to its stable molecular structure and unique redox reaction mechanism (as discussed below).

The galvanostatic intermittent titration technique (GITT) is carried out to evaluate the reaction kinetics of the PTT-4 cathode at a low temperature (Fig. S26). During the charge process, the insertion of the first Al-complex ion [state of charge (SOC) below 50%] into PTT-4 shows relatively fast kinetics with a diffusion coefficient (D) in the order of 10^−9^ cm^2^ s^−1^, while the D of the second ion (SOC 50%–100%) is smaller at 10^−9^–10^−11^ cm^2^ s^−1^. During the discharge process, the first ion extracts quickly from PTT-4 with D ∼ 10^−9^ cm^2^ s^−1^ (SOC 100%–50%), and the second ion slightly more slowly in the same order (SOC below 50%). These values indicate the fast diffusion of Al-complex ions within the porous polymer cathode, which is comparable to that of AlCl_4_^−^ ions in graphite cathodes (10^−9^–10^−11^ cm^2^ s^−1^) [37].

### Redox reaction mechanism of PTT-4 cathode

**Figure 4.**
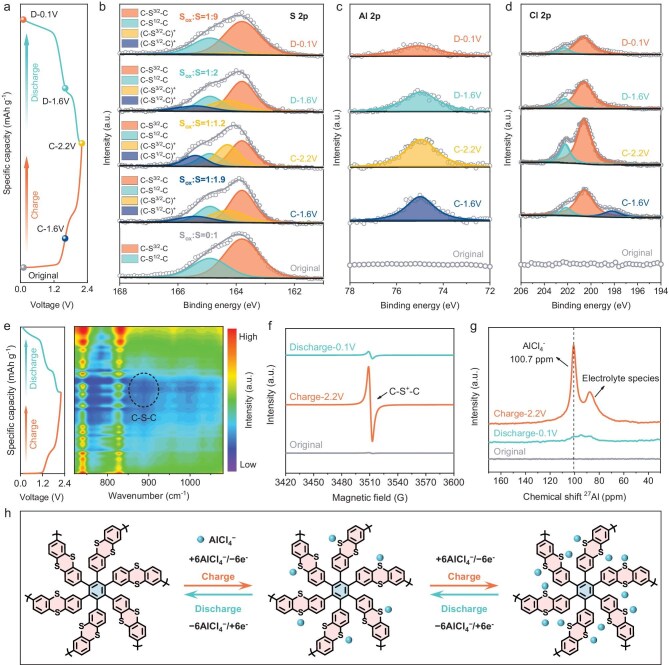
Redox reaction mechanism of PTT-4 cathode. (a) The charge/discharge curves of the PTT-4 cathode and the selected charge/discharge states. *Ex situ* XPS spectra of (b) S 2p, (c) Al 2p and (d) Cl 2p. (a–d) C, charge; D, discharge. (e) *In situ* FTIR spectra. (f) *Ex situ* EPR spectra. (g) *Ex situ* SS ^27^Al NMR spectra. (h) The proposed two-step 12-electron-transfer redox reaction mechanism of PTT-4.

A series of *in situ* and *ex situ* characterizations are performed to clarify the electrochemical reaction mechanism of the PTT-4 cathode. To identify the active sites of PTT-4, *ex situ* XPS spectra on the electrodes at different charge/discharge states are shown in Fig. 4a. The full survey XPS spectra show three peaks located at 164, 285 and 688 eV, corresponding to S 2p, C 1s and F 1s, respectively (Fig. S27). Notably, during the charge process, the S 2p peak migrates to the high-energy region due to the oxidation of neutral thioether (C‒S‒C) groups to C‒S^+^‒C cationic radicals [30,38], and then returns to the low-energy region during the discharge process. To further reveal this process, we investigate the high-resolution S 2p XPS spectra of the PTT-4 cathode (Fig. 4b). The S 2p peak of the original electrode is fitted into two peaks at 163.8 and 164.9 eV, corresponding to the neutral C‒S^3/2^‒C and C‒S^1/2^‒C groups, respectively [39]. During the charge process, two new broad peaks appear at the higher binding energies of 164.3 and 165.4 eV, which can be assigned to the oxidized (C‒S^3/2^‒C)^+^ and (C‒S^1/2^‒C)^+^ radicals [30,39]. During the discharge process, these two peaks decrease significantly due to the reduction of C‒S^+^‒C cationic radicals. Moreover, the calculated ratio of oxidized to neutral S species increases from 0:1 to 1:1.2 after fully charging to 2.2 V, and then decreases to 1:9 after fully discharging to 0.1 V, indicating high redox reversibility. It is noted that partial neutral S species can still be detected after fully charging to 2.2 V, which may be ascribed to the activation process in the first few cycles and incomplete oxidation of C‒S‒C groups. Furthermore, Al 2p and Cl 2p XPS spectra are used to identify the type of active ions involved in the redox reaction (Fig. 4c and d). The peak intensities of Al 2p and Cl 2p increase significantly during the charge process, indicating that an Al-complex anion is inserted into the PTT-4 cathode. On the contrary, the peak intensity decreases during the discharge process, suggesting the reversible extraction of the Al-complex anion from PTT-4. The weak Al and Cl signals detected in the ‘D-0.1 V’ state are related to the residual electrolyte. These results indicate that the C‒S‒C groups in PTT-4 are the active sites involved in the redox reaction. During the charge process, the neutral C‒S‒C groups are oxidized to C‒S^+^‒C cationic radicals and coordinate with Al-complex anions, while the discharge process is reversible.

To further reveal the redox reversibility of PTT-4, *in situ* FTIR spectra are performed to detect its structural evolution during charge and discharge processes (Fig. 4e). During the charge process, the signal intensity of C‒S‒C groups at about 886 cm^−1^ gradually decrease, indicating that the S atoms lose electrons and participate in the redox reaction. During the discharge process, the C‒S‒C signal recovers, demonstrating high redox reversibility. The formation of C‒S^+^‒C cationic radicals in the PTT-4 cathode is further confirmed by *ex situ* electron paramagnetic resonance (EPR). As shown in Fig. 4f, the EPR signal of the original electrode is almost undetectable. After fully charging to 2.2 V, an intense EPR signal appears, indicating the formation of C‒S^+^‒C radicals due to the loss of electrons from S atoms [31]. The corresponding spectral splitting factor *g* is 2.003, suggesting that C‒S^+^‒C is the persistent radical (Fig. S28). After fully discharging to 0.1 V, the significantly weakened EPR signal indicates the reversible reduction of C‒S^+^‒C radicals to C‒S‒C groups. Generally, due to the conjugation effect of adjacent benzene rings, the generated cationic radicals on the sulfur heterocycle can be stabilized by electron delocalization along the molecular skeleton [40]. Such a p-type redox mechanism does not usually involve bond breaking and rearrangement, ensuring fast redox kinetics and high structural stability [15,26].

To further clarify the active anion species at the molecular level, we carried out solid-state ^27^Al single-pulse magic-angle-spinning NMR (SS ^27^Al MAS NMR) tests on fully charged and discharged PTT-4 cathodes. As shown in Fig. 4g, there is no ^27^Al signal in the original PTT-4 electrode. After fully charging to 2.2 V, an intense AlCl_4_^−^ signal at 100.7 ppm and a weak signal of residual electrolyte are observed [28,41], indicating that the active anion is mainly AlCl_4_^−^. After fully discharging to 0.1 V, the ^27^Al signal decreases significantly, implying the reversible dissociation of AlCl_4_^−^ anions from PTT-4. Moreover, the EDS mapping also reveals the type of active anions (Figs S29 and S30). After fully charging to 2.2 V, the Cl/Al atomic ratio in the PTT-4 cathode is 3.88, indicating that the active ion is AlCl_4_^−^ [25]. After fully discharging to 0.1 V, the Cl/Al atomic ratio decreases to 1.67, which is related to the residual electrolyte.

As a result, a two-step 12-electron-transfer redox reaction mechanism is proposed for PTT-4, with 6-electron-transfer for each step (Fig. 4h). During the charge process, two C‒S‒C groups of each TT unit are oxidized sequentially to C‒S^+^‒C cationic radicals, and simultaneously coordinate with two AlCl_4_^−^ anions. During the discharge process, the oxidized C‒S^+^‒C radicals undergo a two-step reduction reaction, accompanied by the reversible dissociation of AlCl_4_^−^ anions. The high-density active C‒S‒C sites and the dual-electron-transfer reaction of TT units contribute to a high capacity and energy density for PTT-4.

### Density functional theory (DFT) calculations

**Figure 5.**
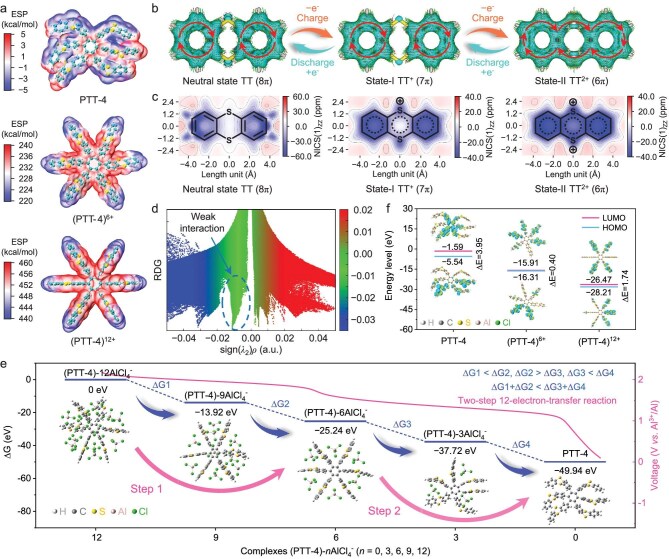
DFT calculations for the 12-electron redox reaction mechanism of PTT-4. (a) Molecular ESP distribution of PTT-4, (PTT-4)^6+^ and (PTT-4)^12+^. (b) AICD plots and (c) NICS(1)zz maps of the neutral TT unit (8 π) and its oxidation states TT^+^ (7 π) and TT^2+^ (6 π). The red arrows in AICD plots refer to the direction of induced current. (d) Scatter plot of RDG vs. sign (*λ*_2_)*ρ* of TT–2AlCl_4_^−^. The blue region represents notable attraction, the green region represents weak interaction, and the red region represents notable repulsion. (e) Calculated ΔG values and the optimized structures of PTT-4 and its complexes (PTT-4)-*n*AlCl_4_^−^ (*n* = 3, 6, 9, 12). (f) Calculated LUMO/HOMO energy levels of PTT-4, (PTT-4)^6+^ and (PTT-4)^12+^.

DFT calculations are performed to further reveal the redox reaction mechanism of PTT-4. Firstly, molecular ESP distribution is calculated to identify the active sites. The neutral PTT-4 shows obvious negative ESP in the sulfur-heterocycle regions of each TT unit, indicating an electron-donating tendency (Fig. 5a). In contrast, the oxidized PTT^6+^ and PTT^12+^ show intensely positive ESP throughout the molecule, especially in the sulfur-heterocycle regions, which can act as the active sites to attract anions. Furthermore, similar ESP distributions can also be observed in PTT-1, PTT-2, PTT-3 and their oxidation-state species, meaning that they have the same active sites and charge storage mechanism (Fig. S31). Moreover, atomic charge analysis is performed to further determine the active sites of PTT-4. Compared with neutral PTT-4, the oxidized PTT^6+^ and PTT^12+^ show more positive Hirshfeld charge (HC) values on the S heteroatoms (Fig. S32), suggesting that the loss of electrons mainly occurs on the S atoms during the oxidation process. These results indicate that the C‒S‒C groups in TT units are the active sites involved in the redox reaction.

Generally, aromatic molecules exhibit prominent structural stability due to the π-electron delocalization effect, which contributes to a long cycling performance [40]. To investigate the structural stability of TT molecule units during the oxidation process, we calculate the anisotropy of the induced current density (AICD) and nucleus-independent chemical shift (NICS) to analyze the aromaticity (Fig. 5b and c) [42]. In the neutral TT molecule, the clockwise continuous current flows over the benzene rings on either side of the S heterocycle, showing strong aromatic characteristic. Notably, there is no net current on the central S heterocycle, indicating a significant non-aromatic characteristic. It should be pointed out that although the S heterocycle has 8 π-electrons, it is not antiaromatic (as predicted by Hückel’s rule), but non-aromatic because of its non-planar conformation [43,44]. This can be also reflected by the NICS(1)zz value (∼0 ppm) of the heterocycle region in the neutral TT molecule. After losing one electron from TT (8 π) to TT^+^ (7 π), the S heterocycle still exhibits non-aromatic characteristics. However, due to the conjugation effect [40], they can be well stabilized by the robust benzene rings with great aromaticity during the redox process. After losing the other electron from TT^+^ (7 π) to TT^2+^ (6 π), the S heterocycle transforms to aromaticity, as suggested by the clockwise circular current throughout the molecule in AICD plots and negative NICS(1)zz values. Such non-aromatic–aromatic transformation of the S heterocycle indicates an enhanced structural stability in the oxidation process, ensuring stable storage of large AlCl_4_^−^ carriers during long cycling.

The π-electron delocalization effect of TT units is further revealed by the localized orbital location-π (LOL-π) method (Fig. S33) [45]. After being fully oxidized to TT^2+^, the whole molecule shows a continuous π-electron delocalization path, indicating strong aromaticity and high structural stability. This enhanced π-electron delocalization effect may be related to the conformation transformation of the TT molecule from non-planar to planar (Fig. S34). Due to the instability of its planar form, the original TT molecule adopts a non-planar geometry conformation, minimizing the total energy [46]. Interestingly, after losing one or two electrons, the oxidized TT^+^ and TT^2+^ show a near-planar or planar conformation, which facilitates the electron delocalization throughout the molecular skeleton. Notably, when the TT units are integrated into PTT-4, they also exhibit improved π-electron delocalization behavior during the oxidation process (Fig. S35). Furthermore, we calculate the electron spin density (ESD) to further reveal the structural stability of the radical intermediate TT^+^ (7 π) (Fig. S36). It is observed that the free radical electrons mainly congregate around the S heteroatoms, which can act as active sites to participate in the redox reaction. More importantly, the electrons are further delocalized to the entire conjugated structure, ensuring the stability of the radical intermediate.

To reveal the interaction between the TT unit and the AlCl_4_^−^ anion, we perform reduced density gradient (RDG) analysis (Fig. 5d) [47]. The detected green spike in sign(*λ*_2_)*ρ* from −0.02 to 0.00 a.u. indicates a weak interaction between the TT unit and the AlCl_4_^−^ anion. Furthermore, the independent gradient model based on Hirshfeld partition (IGMH) analysis [48] indicates that the AlCl_4_^−^ anions tend to generate weak interaction with two adjacent TT units in PTT-4 (Fig. S37). In addition, the electron density difference (EDD) plots further reveal the charge transfer between the TT unit and the AlCl_4_^−^ anion (Fig. S38). When TT coordinates with an AlCl_4_^−^ anion, an electron depletion region (blue area) obviously presents around the S heteroatoms, indicating that the S atoms participate in the electron donating process. It is noted that there is no significant electron transfer from TT to AlCl_4_^−^, suggesting a weak coordination interaction. Such weak interaction between the TT unit and the AlCl_4_^−^ anion does not involve the breaking and formation of chemical bonds, which allows for fast reaction kinetics and guarantees high structural stability during long cycling.

To confirm the two-step 12-electron reaction mechanism, we calculate the Gibbs free energy differences (ΔG) of the discharge process (Fig. 5e and Table S5). Considering the symmetry of the molecular structure of PTT-4, we construct four possible complex configurations (PTT-4)-*n*AlCl_4_^−^ (*n* = 3, 6, 9, 12). The calculated ΔG of the first step (discharge reaction) is smaller than that of the second step (ΔG_1_ < ΔG_2_), indicating that the first two-step reactions can occur successively. Similarly, the last two-step reactions can also take place spontaneously (ΔG_3_ < ΔG_4_). However, the ΔG of each step from ΔG_1_ to ΔG_4_ does not increase continuously, implying that a continuous four-step discharge process may not occur [40]. Notably, the sum of ΔG_1_ + ΔG_2_ is smaller than that of ΔG_3_ + ΔG_4_, indicating that the whole discharge process can be divided into two steps, which is consistent with the two discharge voltage plateaus. As a result, a two-step 12-electron-transfer reaction mechanism is demonstrated for PTT-4, with 6-electron-transfer for each step. In other words, each TT unit undergoes a two-step dual-electron-transfer reaction, and coordinates with two AlCl_4_^−^ anions.

To further clarify the structure stability of PTT-4 during the reaction process, we calculate the LUMO/HOMO energy levels of PTT-4 at different oxidation states (Fig. 5f). The frontier electrons of PTT-4, (PTT-4)^6+^ and (PTT-4)^12+^ are mainly distributed on the TT units, and are well retained in the molecular structure, indicating that all the oxidation states are stable. This means that the PTT-4 can carry up to 12 positive charges. Moreover, compared with neutral PTT-4, the oxidized (PTT-4)^6+^ and (PTT-4)^12+^ display smaller energy gaps (∆E), showing enhanced electron transfer ability, which is conducive to binding with anions during the charge process. Furthermore, the calculated ∆E of (PTT-4)–6AlCl_4_^−^ and (PTT-4)–12AlCl_4_^−^ complexes is also smaller than that of PTT-4 (Fig. S39), further demonstrating the thermodynamically favorable reaction between PTT-4 and AlCl_4_^−^ anions [21,26].

## CONCLUSIONS

Designing high-voltage and high-capacity organic cathode materials with rational molecular structure is crucial for attaining high-energy and long-life AOBs. However, the energy density of most organic cathodes is restricted to the range of conventional graphite cathodes (100−200 Wh kg^−1^), based on the mass of cathode materials. We have demonstrated a promising linking-patterns-tailoring strategy for designing a multisite super-crosslinked sulfur-heterocyclic polymer cathode with a weak electron-donating effect to maximize both the operating voltage and specific capacity. This molecular tailoring strategy substantially promotes the redox activity and achieves 12-electron-transfer, resulting in a high voltage of up to 2.0 V (average ∼1.7 V) and a high capacity of 150 mAh g^−1^, corresponding to an energy density of 255 Wh kg^−1^. Notably, the constructed AOB with a PTT-4 cathode exhibits extraordinary cycling stability at low temperature, with almost no capacity degradation, even after 12 000 cycles at −20°C. The non-planar–planar conformation transformation of TT active units and weak coordination interaction between C‒S^+^‒C cationic radicals and AlCl_4_^−^ anionic carriers play an important role in preserving the stability of the molecular structure during long-term redox.

Such earth-abundant (C, H, S) organic cathode material with industrial-scale production potential shows an appealing pathway for addressing affordable large-scale energy storage. Whereas these results represent a major advance in the design of AOBs, the limited utilization rate of active sites (<70%) is indicative of a great upward potential of multisite organic cathodes, which is related to incomplete electron transfer. It is undeniable that the electronic conductivity of organic materials still has difficulty competing with that of highly conductive graphite or graphene materials. Future investigations on improving the inherent electronic properties of organic cathodes by introducing conductive units, extending π-conjugated structures and designing electron donor–acceptor structures, are supposed to further improve the battery performance. This work highlights the importance of molecular design for high-energy and low-temperature AOBs, and promotes the development of long-life organic batteries, especially those operating under extreme conditions.

## Supplementary Material

nwaf526_Supplemental_Files
