# Supplementary material for: A multisite super-crosslinked sulfur-heterocyclic polymer cathode for high-voltage and low-temperature aluminum–organic batteries
Source: Natl Sci Rev. 2025 Nov 22;13(1):nwaf526. doi: 10.1093/nsr/nwaf526 (PMC12796798; doi:10.1093/nsr/nwaf526)
Supplement: nwaf526_Supplemental_Files [file nwaf526_supplemental_files.zip › Supplementary data.pdf]

Supporting Information

A multisite super-crosslinked sulfur-heterocyclic polymer cathode for high-voltage and low-temperature aluminum-organic batteries

Yuxi Guo[1,2], Ke Guo[2], Wei Wang[1]*, Zheng Huang[1], Yaxue Wang[1], Mingyong Wang[1], Yanli Zhu[2,]*, and Shuqiang Jiao[1,]*

[1]State Key Laboratory of Advanced Metallurgy, University of Science and Technology Beijing, Beijing 100083, China;

[2]State Key Laboratory of Explosion Science and Safety Protection, Beijing Institute of Technology, Beijing 100081, China.

***Corresponding authors**. E-mails: wwang@ustb.edu.cn; zhuyanli1999@bit.edu.cn; sjiao@ustb.edu.cn

Experimental Section

Materials

Dihydrophenazine (PZ, 95%), phenothiazine (PTZ, 98%), phenoxazine (PXZ, 97%), thianthrene (TT, 98%), 1,4-dibromobenzene (99%), 1,3,5-tribromobenzene (98%), 1,2,4,5-tetrabromobenzene (97%), hexabromobenzene (99%), bis(pinacolato)diboron (98%), sodium hydrogen carbonate ($NaHCO_3$, 99.5%), anhydrous potassium carbonate (K_2CO_3, 99%), Tetrakis(triphenylphosphine)palladium ($Pd(PPh_3)_4$, 99%), anhydrous aluminum chloride ($AlCl_3$, 99.9%) and 1-ethyl-3-methylimidazolium chloride ([EMIm]Cl, 98.0%), acetic acid (AcOH, 99.5%), N,N-dimethylformamide (DMF, 99.5%), dichloromethane (DCM, 99.5%), methanol (MeOH, 99.5%) and N-methyl-2-pyrrolidinone (NMP, 99.5%) were purchased from Macklin (Shanghai, China). Bromine (Br_2, 99.5%) was purchased from Sinopharm Chemical Reagent Co., LTD (Shanghai, China). Aluminum foil (50 μm thickness, 99.99%) and tantalum foil (10 μm thickness, 99.95%) were purchased from Qing Yuan metal co. LTD (China). Molybdenum mesh (20 μm thickness, 100 mesh, 99.99%) was purchased from Guan Tai metal co. LTD (China). All materials were used directly without further purification or treatment.

Materials synthesis

Synthesis of 2,8-dibromothianthrene (TT-Br$_2$)

In a 100 mL round-bottom flask, thianthrene (2.16 g, 10 mmol) and bromine (4 mL) were slowly added to acetic acid (40 mL). The solution was refluxed at 80 °C for 16 h. After the reaction, deionized water (20 mL) was added to obtain a white solid. The mixture was filtered and washed with $NaHCO_3$ solution (5%), then recrystallized with MeOH and DCM to obtain a white powder TT-Br$_2$ (3.2 g, yield: 86%).

Synthesis of 2,8-bis(4,4,5,5-tetramethyl-1,3,2-dioxaborolan-2-yl)-thianthrene (TT-BO$_2$)

Under N_2 atmosphere, TT-Br$_2$ (3.74 g, 10 mmol), bis(pinacolato)diboron (6.47 g, 25.5 mmol), potassium acetate (3.0 g, 30.5 mmol) and Pd(dppf)Cl_2 (0.15 g, 0.2 mmol) were added into 1,4-dioxane

(100 mL) The reaction solution was heated to 90 °C and stirred for 48 hours. After cooling to room temperature, the reaction was quenched by water (50 mL). Then, the mixture was extracted by DCM for three times (3×20 mL). The combined organic layer was washed with deionized water (3×50 mL), then dried over $MgSO_4$ and the solvent was removed by vacuum. The crude product was purified by column chromatography on silica gel to obtain a brown powder TT-BO_2 (4.45 g, yield: 95%).

Synthesis of sulfur-heterocyclic polymers (PTT-n, n=1, 2, 3, 4)

PTT-n (n=1, 2, 3, 4) was synthesized according to a previously reported method with modifications [1,2]. Under N_2 atmosphere, TT-BO_2 (0.468 g, 1 mmol), hexabromobenzene (0.184 g, 0.33 mmol), K_2CO_3 (1.1 g, 5.26 mmol), and $Pd(PPh_3)_4$ (0.04g, 0.035 mmol) were added to a mixture of DMF (16 mL) and deionized water (4 mL) in a flame-dried Schlenk tube. The reaction solution was heated to 150 °C and stirred for 48 hours. After the reaction, the product was filtered and rinsed with DMF, deionized water and methanol to remove the residual reagents. Then, the solid was dried at 80 °C for 24 h under vacuum to obtain a gray powder PTT-4 (0.224 g, yield: 90%). PTT-1, PTT-2 and PTT-3 were synthesized using the same method by replacing hexabromobenzene with 1,4-dibromobenzene (0.236 g, 1 mmol), 1,3,5-tribromobenzene (0.21 g, 0.667 mmol) and 1,2,4,5-tetrabromobenzene (0.197 g, 0.5 mmol), respectively.

Material characterizations

Scanning electron microscope (SEM, Zeiss Gemini 300) and energy-dispersive spectroscopy (EDS) were used to characterized the morphology of and elemental mapping of samples. ^{1}H and ^{13}C Nuclear magnetic resonance (NMR) spectra were recorded on a Bruker 400M NMR spectrometer with $CDCl_3$ as the solvent. Fourier transform infrared (FTIR) spectra were conducted on Thermo Fisher Scientific Nicolet iS20 spectrometer from 400 to 4000 cm^{-1}. In-situ electrochemical FTIR test was performed on Thermo IS50 spectrometer from 650 to 4000 cm^{-1}. Solid-state ^{13}C and ^{27}Al NMR measurements

were performed at a MAS rate of 10 kHz on a Bruker 400M NMR spectrometer. X-ray photoelectron spectroscopy (XPS) was obtained on a Thermo Scientific K-Alpha spectrometer with Al Kα (1486.6 eV) as the excitation source. X-ray diffraction (XRD) patterns were collected using a Rigaku, D/max-RB X-ray diffractometer with a Cu Kα radiation (λ=1.5406 Å). N_2 adsorption-desorption analysis was carried out on a Micromeritics Tristar II 3020 BET analyzer at 80 °C. The specific surface area and size distribution of PTT were estimated by Brunauer–Emmett–Teller (BET) and DFT methods, respectively. Thermogravimetric analysis (TGA) was performed on a Rigaku TG/DTA8122 thermal analyzer at a heating rate of 10 °C min^{-1} under N_2 flow. The ultraviolet visible (UV-Vis) spectra were collected on a hitachi UH4150 spectrometer.

Electron paramagnetic resonance (EPR) was carried out on a Bruker ELEXSYS-II E500 spectrometer. To compare the signals intensity of the prepared ex-situ electrodes, 5 mg powder samples were weighed for quantitative testing. According to Zeeman effect, under a resonance magnetic field, the electron orbital can split into two suborbitals and the energy difference between the two suborbitals is $\Delta E = g\beta H$. The relationship between spectral splitting factor (g-factor) and magnetic field strength (H) can be calculated through the following equation:

$$g = \frac{hv}{\beta H} \quad (1)$$

where h is Planck constant (6.626×10^{-34} J·s), β is Bohr magneton (9.274×10^{-24} J·T^{-1}), v is the microwave frequency (9.843 GHz).

Electrochemical measurements

The electrochemical performances were evaluated using Swagelok cells and pouch batteries. To prepare the organic cathode, active material (TT monomer and PTT polymers), acetylene black and polyvinylidene fluoride (PVDF) binder were mixed homogeneously in NMP by a weight ratio of 6:3:1, then the resulting slurry was cast onto on the tantalum (Ta) foil (thickness: 10 μm), followed by drying overnight at 60 °C under vacuum. The mass loading of active materials is ca. 1.0–1.5 mg cm^{-2}. To

prepare the ionic liquid electrolyte, $AlCl_3$ was slowly added to [EMIm]Cl at a molar ratio of 1.3:1 ($AlCl_3$:[EMIm]Cl) in an argon-filled glove box ($O_2 < 0.1$ ppm, $H_2O < 0.1$ ppm). Swagelok-type cells were assembled with an organic cathode (diameter: 12 mm), a glass fiber separator (Whatman GF/A, 14 mm), an aluminum foil anode (diameter: 14 mm), and $AlCl_3$/[EMIm]Cl electrolyte (100 μL) in the argon-filled glove box. For in-situ electrochemical FTIR test, the PTT-4 organic cathode was prepared using the same method with molybdenum (Mo) mesh as the current collector. The cell was assembled with a PTT-4 cathode (diameter: 12 mm), a glass fiber separator (Whatman GF/A, diameter: 18 mm), an aluminum foil anode (diameter: 14 mm), and 150 μL $AlCl_3$/[EMIm]Cl electrolyte in an in-situ electrochemical test cell with an infrared window. The Al||PTT-4 pouch-type batteries were assembled with two layers of PTT-4 cathode (5×8 cm), two layers of glass fiber separators (Whatman GF/A, 7×10 cm), one layer of Al anode (6×9 cm) and 6 mL $AlCl_3$/[EMIm]Cl electrolyte.

Galvanostatic charge/discharge tests were performed on the multichannel battery testing system (Neware BTS-53) in a voltage range of 0.1–2.2 V at 25 °C. Cyclic voltammetry (CV) tests were conducted on EC-Lab electrochemical workstation in a voltage range of 0.1–2.3 V at different scan rates. Electrochemical impedance spectroscopy (EIS) tests were carried out in a frequency range of 100 kHz to 0.01 Hz with a perturbation amplitude of 5 mV under the open-circuit potential. Galvanostatic intermittent titration technique (GITT) tests were performed with a galvanostatic pulse current 0.2 A g^{-1} for 2 min followed by an open-circuit relaxation of 1 h.

Density functional theory calculations

All calculations were performed using Gaussian 16 software Package [3]. The geometry structures were optimized at the level of B3LYP [4]/def2-SVP [5] with Grimme's DFT-D3(BJ) empirical dispersion correction [4]. The vibration frequency calculations were also performed at the same level to confirm that the optimized structure is the local minimum point on the potential energy surface without imaginary frequency. The thermal correction of Gibbs free energy was obtained from

frequency calculations. We further calculate the precise single point energy at a higher level of B3LYP-D3/def2-TZVP [5]. The Gibbs free energy (G) was calculated using the Shermo program [6] based on the precise single point energy, the thermal correction value of Gibbs free energy, and the zero point energy (ZPE) correction factor. According to the ZPVE15/10 database [7], the ZPE correction factor of the B3LYP/def2-SVP level was fitted as 0.9826. The Gibbs free energy differences (ΔG) of the discharge process of PTT-4 were calculated based on the equation:

$$\Delta G = G_f - G_i - nG_{AlCl_4^-} \quad (2)$$

where G_f, G_i and $G_{AlCl_4^-}$ are the sum of electronic and the correction of thermal Gibbs free energy for the final and initial state of PTT-4 complexes, and $AlCl_4^-$ ion, respectively; n is the electron transfer number.

The calculated results of molecular electrostatic potential (ESP), molecular orbital composition, reduced density gradient (RDG), independent gradient model based on Hirshfeld partition (IGMH), nucleus-independent chemical shift (NICS), localized orbital location-π (LOL-π), electron density difference (EDD), electronic spin density (ESD), and Hirshfeld charges (HCs) were analyzed using Multiwfn 3.8 programs [8]. The visualization of all isosurface maps were carried out by Visual Molecule Dynamics (VMD 1.9.3) software [9]. The anisotropy of the induced current density (AICD) were calculated by AICD program [10].

Evaluation of the theoretical capacity and energy density

The theoretical capacity of PTT-n (n=1, 2, 3, 4) was calculated through the following equation:

$$C_{capacity} = \frac{n \times F}{3.6M} \quad (3)$$

where *n* is the number of electron transfer, *F* is Faraday's constant (96485.33 C mol^{-1}), M is the relative molecular weight of PTT-n (n=1, 2, 3, 4). The mole ratios of active TT units (M_{TT} = 216 g mol^{-1}) and aromatic benzene linkers (M_{Bz} = 78 g mol^{-1}) in PTT-1, PTT-2, PTT-3 and PTT-4 are 1:1, 3:2, 2:1 and 3:1, respectively. Therefore, the molecular weight of each repeating structure unit for 2-electrons

transfer is calculated to be M_{PTT-1} = 214+76 = 290 g mol^{-1}, M_{PTT-2} = 214+75×2/3 = 264 g mol^{-1}, M_{PTT-3} = 214+74×1/2 = 251 g mol^{-1} and M_{PTT-4} = 214+72×1/3 = 238 g mol^{-1}, respectively. Based on equation 2, the calculated theoretical capacities of PTT-1, PTT-2, PTT-3 and PTT-4 are 184.8, 203, 213.6 and 225.2 mAh g^{-1}, respectively (table S1).

The energy density of aluminum-organic batteries was calculated as follows:

$$E = \frac{VC}{m} \quad (4)$$

where E is the energy density (Wh kg^{-1}), V is the average discharge voltage (V) of the battery, C is the capacity (mAh), m is the mass (mg) of the active material. Therefore, the energy density of the Al||PTT-4 battery based on the mass of PTT-4 reaches 255 Wh kg^{-1}, which is higher than those reported in most literature for organic and graphitic positive electrode materials in AIBs.

Calculation of the optical energy gap (E_g) from UV-vis test

The optical energy gaps (E_g, eV) of PTT-n (n=1, 2, 3, 4) can be determined by the ultraviolet-visible (UV-Vis) spectroscopy, which is expressed as [11]:

$$\alpha = \frac{(hv - E_g)^{1/2}}{hv} \quad (5)$$

$$hv = 1208/\lambda \quad (6)$$

where α is the optical absorption coefficient, hv is the photon energy, λ is the wavelength.

Calculation of the activation energy (E_a) from EIS test

The activation energy (E_a, kJ mol^{-1}) for charge transfer was calculated according to the EIS spectra at different temperature, which can be calculated by the Arrhenius equation [11]:

$$1/R_{ct} = A\, exp(-E_a/RT) \quad (7)$$

where R_{ct} represents the charge transfer resistance (Ω), A is a constant under a stable experimental condition, R is the gas constant (8.314 J mol^{-1} K^{-1}), T is the testing temperature (K). The E_a values were obtained by the linear fitting of the ln (R_{ct}^{-1}) vs. 1000/T plots:

$$ln\left({R_{ct}}^{-1}\right) = -Ea/RT + k \quad (8)$$

where k is a constant.

Calculation of the ion diffusion coefficient (D) from GITT test

The ion diffusion coefficient (D) was measured by GITT and calculated based on the following equation:

$$D = \frac{4}{\pi\tau}\left(\frac{n_m V_m}{S}\right)^2\left(\frac{\Delta E_s}{\Delta E_t}\right)^2 \quad (9)$$

Where t and τ represent the duration of current pulse (s) and relaxation time (s), respectively. n_m, V_m and S are the mole number (mol), molar volume (cm^3 mol^{-1}) and electrode area (cm^2) of the active material. ΔE_s and ΔE_t are the voltage change (V) caused by current pulse and the voltage change (V) caused by galvanostatic discharge/charge, respectively.

Analysis of the capacity contribution from CV test

The capacitive and faradaic contributions are determined by fitting the relationship of peak current (i) and scan rate (v) according to the following Equation:

$$i = av^b$$

where b = 0.5, the capacity is contributed by faradaic process (ion diffusion), whereas b = 1 the capacity is contributed by capacitive process. The calculated b values of the redox peaks (O1, O2, R1, R2) are 0.95, 0.86, 0.89, and 0.91, respectively (Fig. S24), indicating a capacitive process.

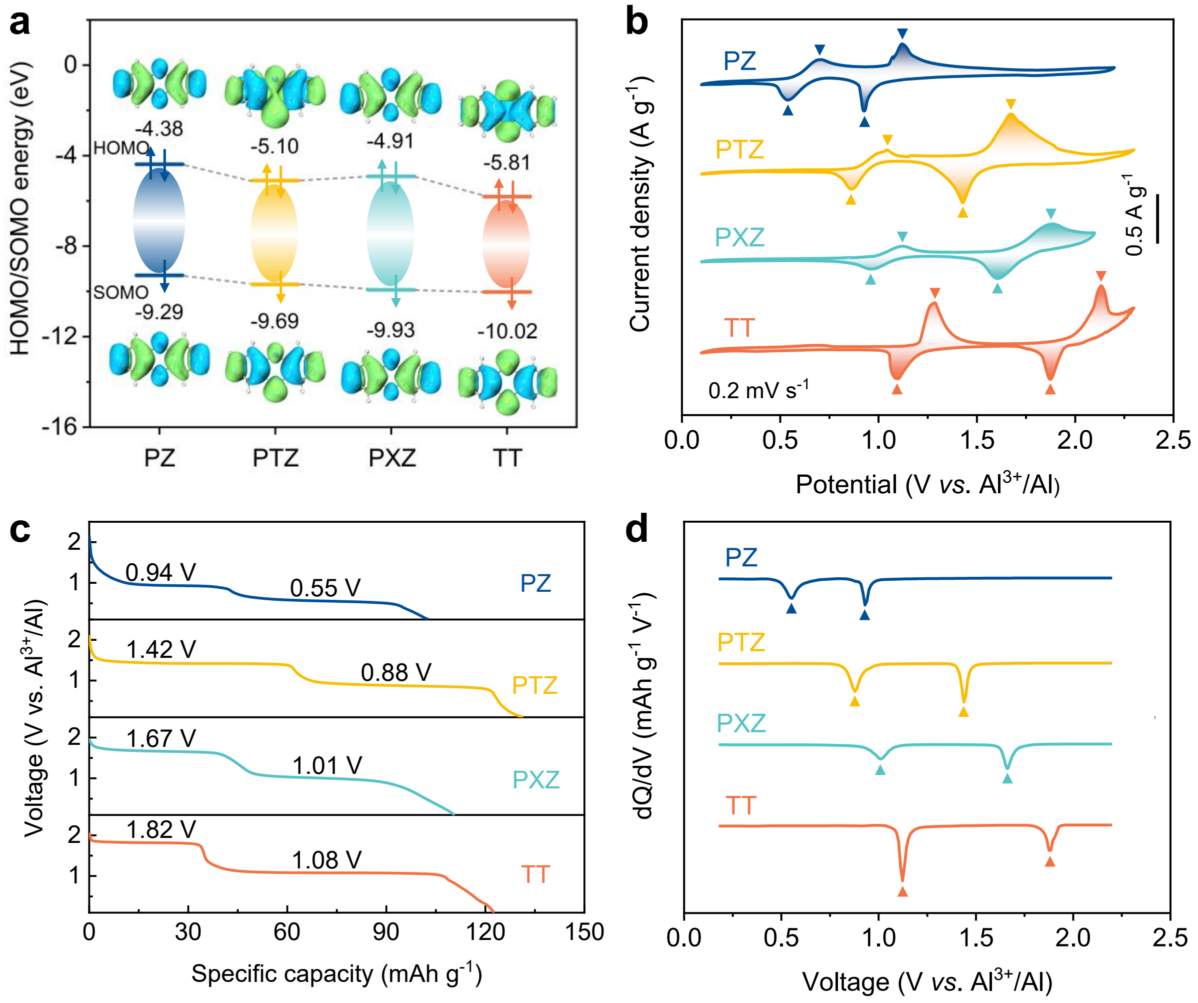

Figure S1. Regulation of the redox potential of conjugated heterocyclic molecules (PZ, PTZ, PXZ, TT). (a) Calculated HOMO/SOMO energy level. (b) CV curves at 0.2 mV s^{-1}. (c) Capacity-voltage profiles at 0.2 A g^{-1} and (d) corresponding differential capacity curves.

Note: The decreased HOMO/SOMO levels reduce the electron-donating effect of conjugated heterocyclic molecules, which leads to higher redox potentials.

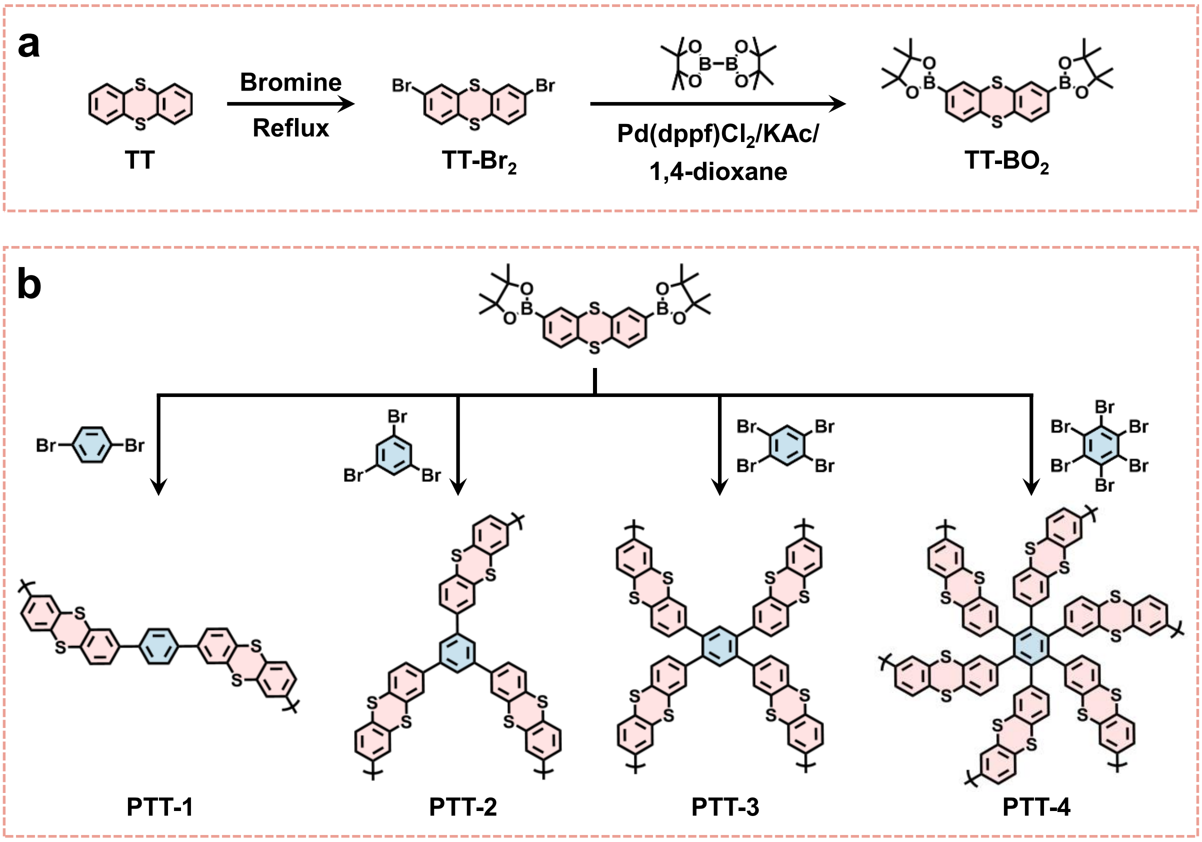

Figure S2. Synthetic route of PTT-n (n=1, 2, 3, 4) polymers. The PTT-n polymers were prepared based on a simple Suzuki coupling reaction.

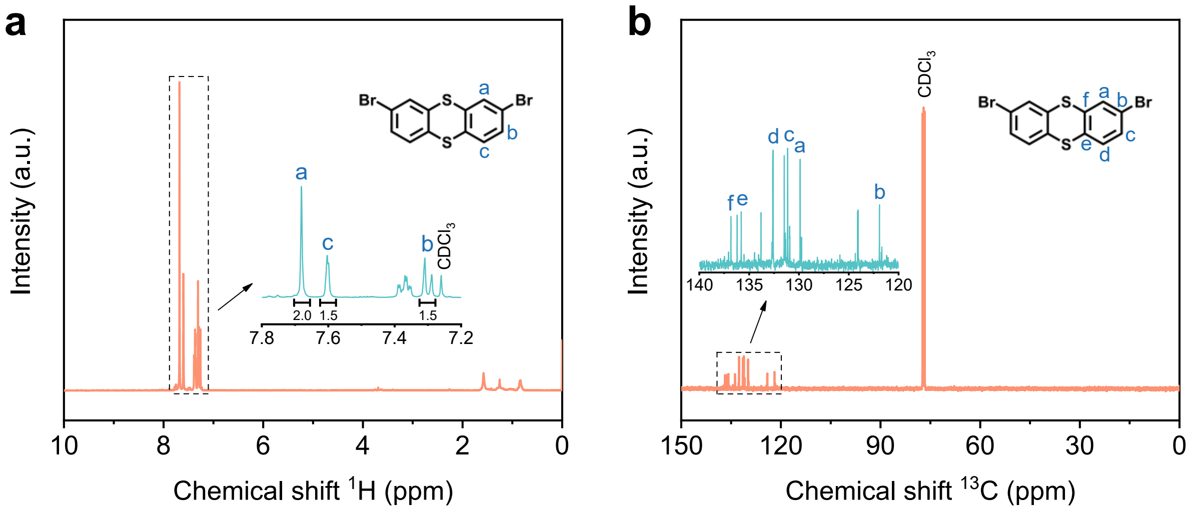

Figure S3. (a) ^{1}H NMR and (b) ^{13}C NMR spectra of TT-Br$_2$ ($CDCl_3$, 400 MHz).

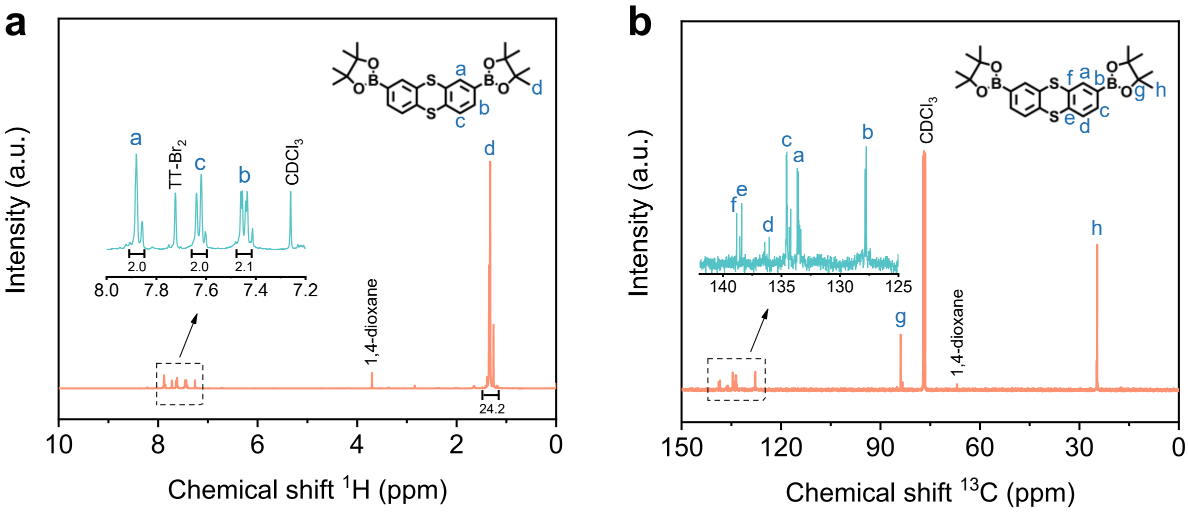

Figure S4. **(a)** ^{1}**H NMR and (b)** ^{13}**C NMR spectra of TT-BO**$_2$ **(CDCl**$_3$**, 400 MHz).**

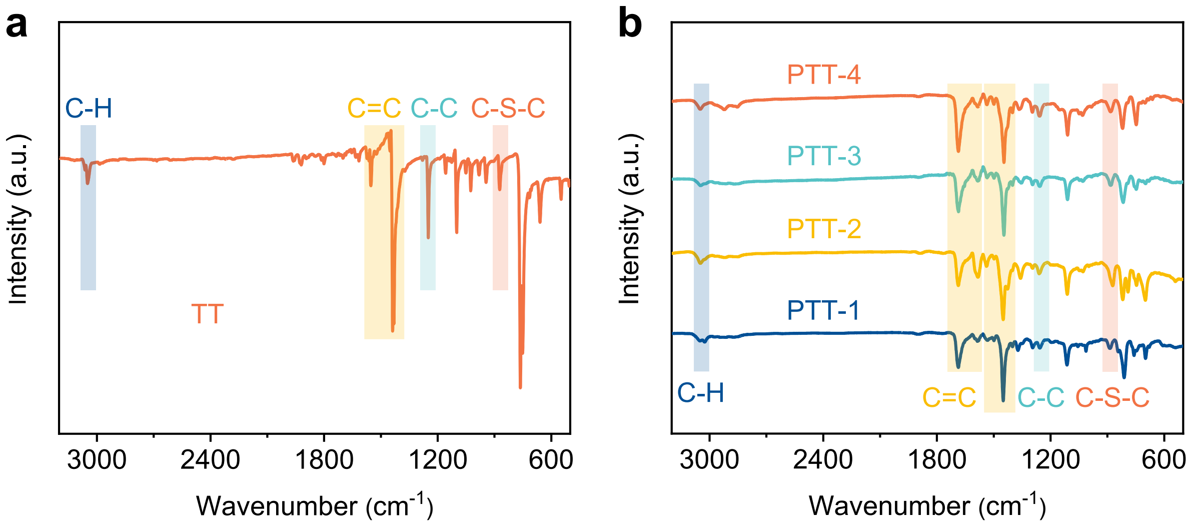

Figure S5. Molecular structure characterization. FTIR spectra of (a) TT monomer and (b) PTT-n (n=1, 2, 3, 4) polymers.

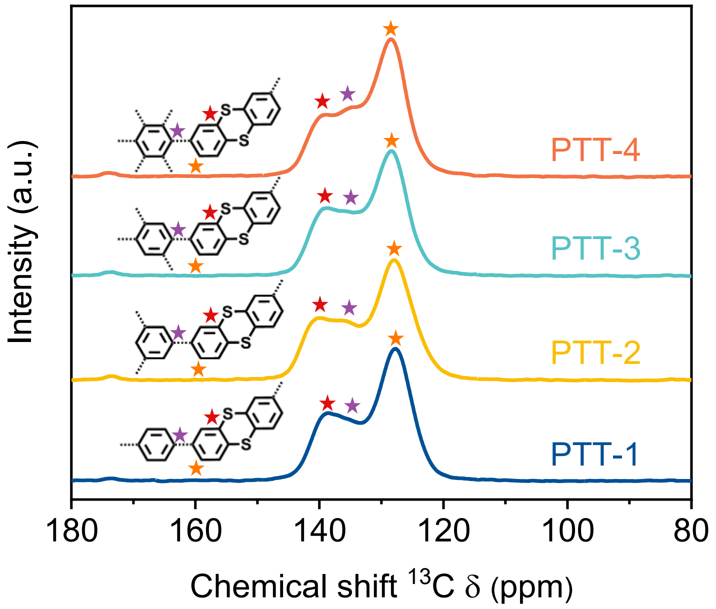

Figure S6. Solid-state ^{13}C NMR spectra of PTT-n (n=1, 2, 3, 4) polymers.

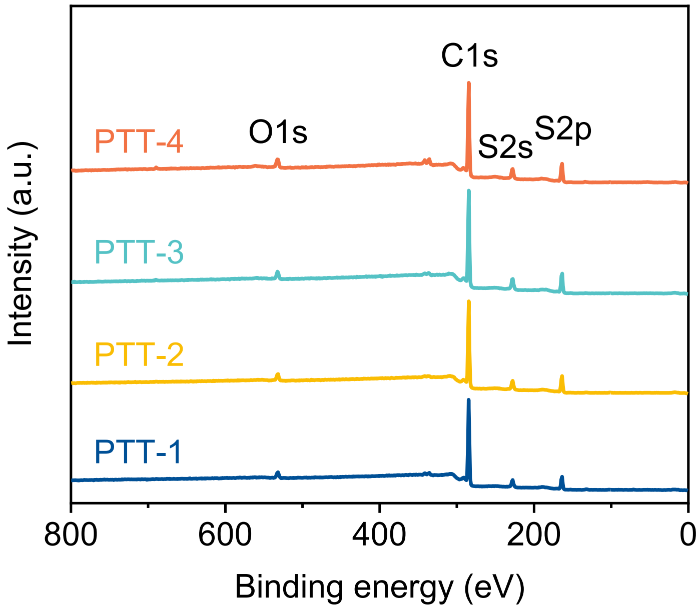

Figure S7. Chemical composition analysis. XPS spectra of PTT-n (n=1, 2, 3, 4) polymers.

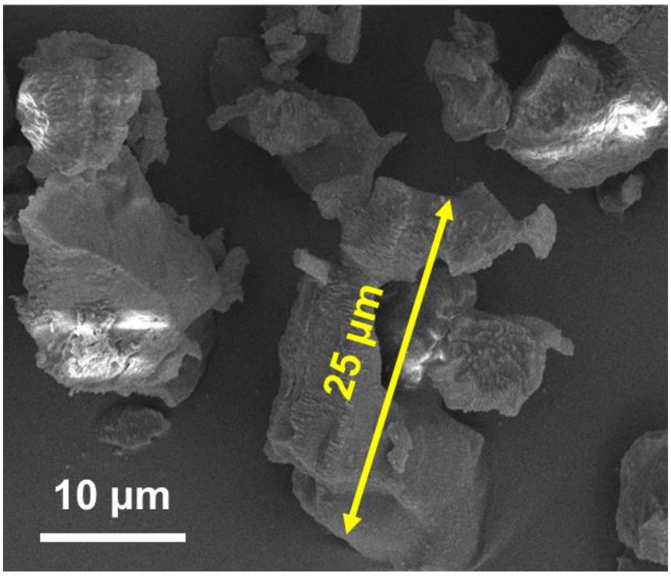

Figure S8. Morphology characterization of TT monomer. SEM image and size of TT micron blocks.

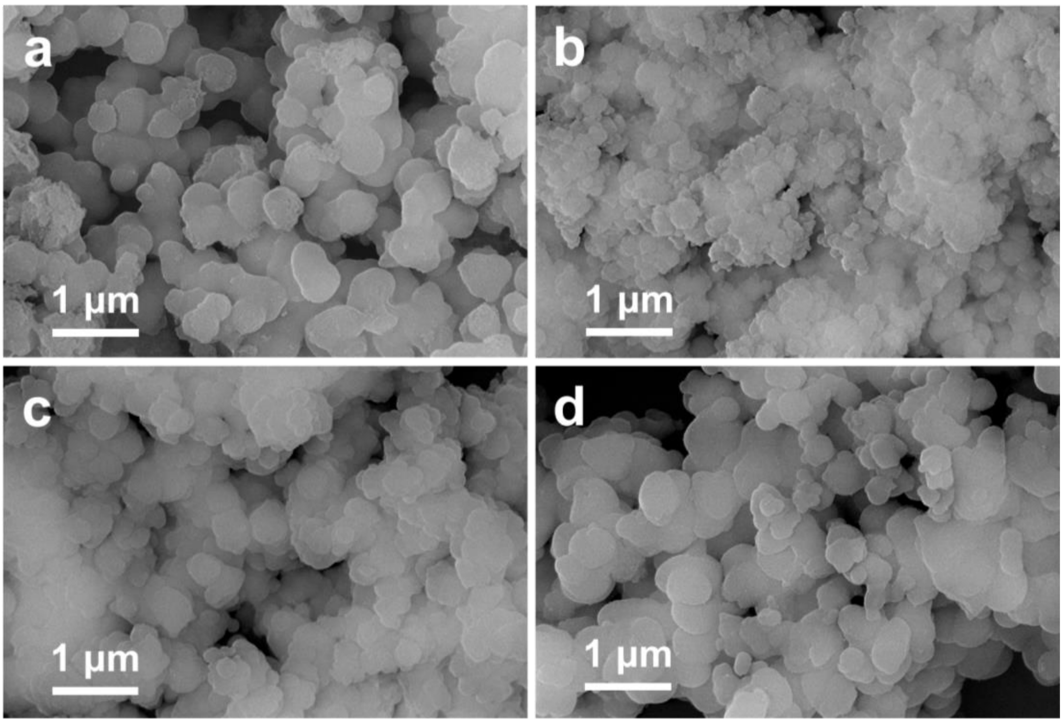

Figure S9. Morphology characterization of PTT-n (n=1, 2, 3, 4) polymers. SEM images of (a) PTT-1, (b) PTT-2, (c) PTT-3, (d) PTT-4.

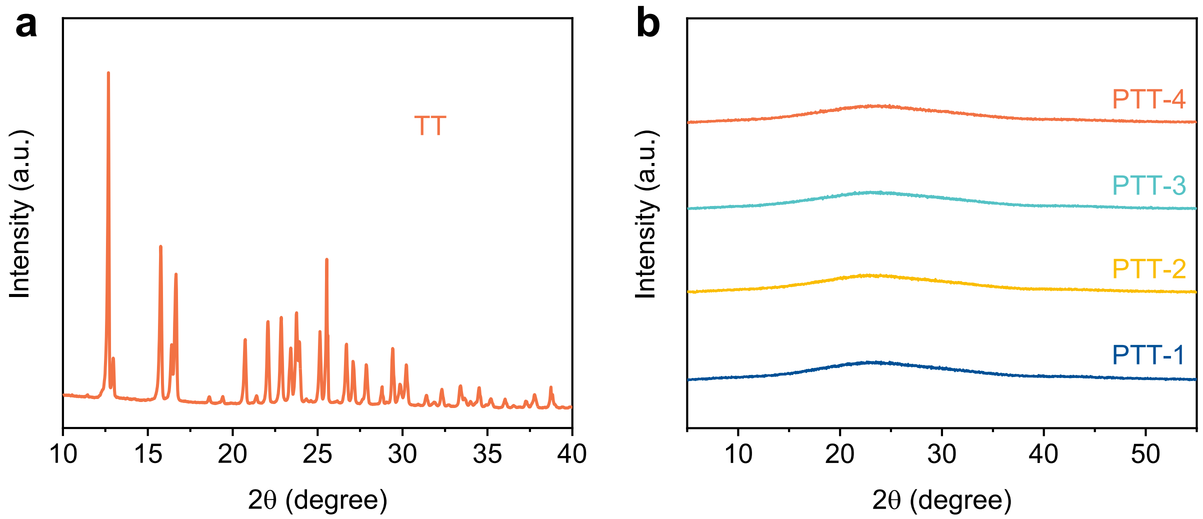

Figure S10. Crystal structure characterization. XRD patterns of (a) TT monomer and (b) PTT-n (n=1, 2, 3, 4) polymers.

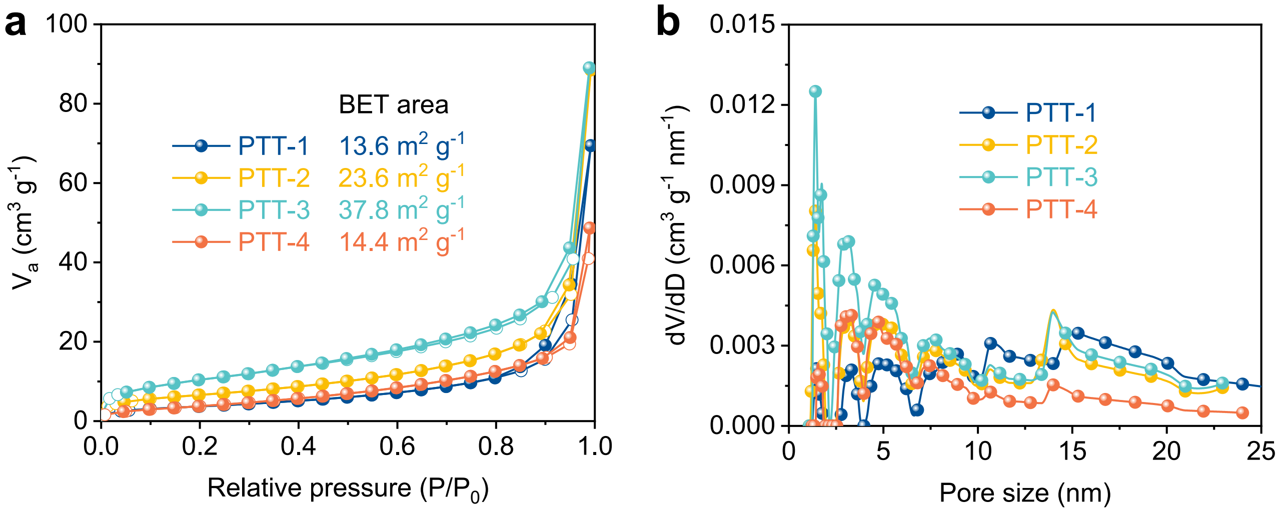

Figure S11. Porous structure characterization of PTT-n (n=1, 2, 3, 4) polymers. (a) N_2 adsorption–desorption isotherms. (b) The pore distribution estimated using nonlocal density functional theory (DFT) method.

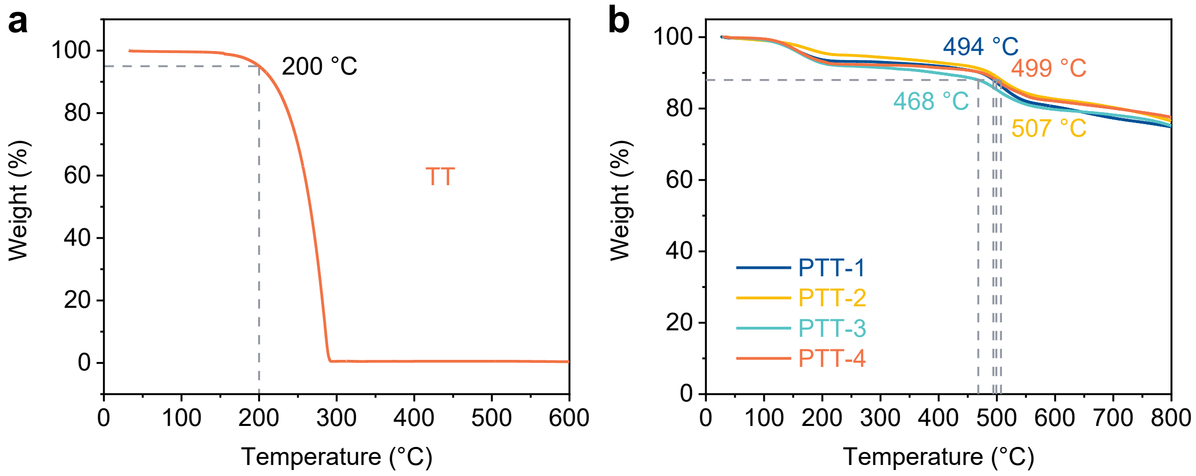

Figure S12. Thermal stability characterization. TGA curves of (a) TT monomer and (b) PTT-n (n=1, 2, 3, 4) polymers.

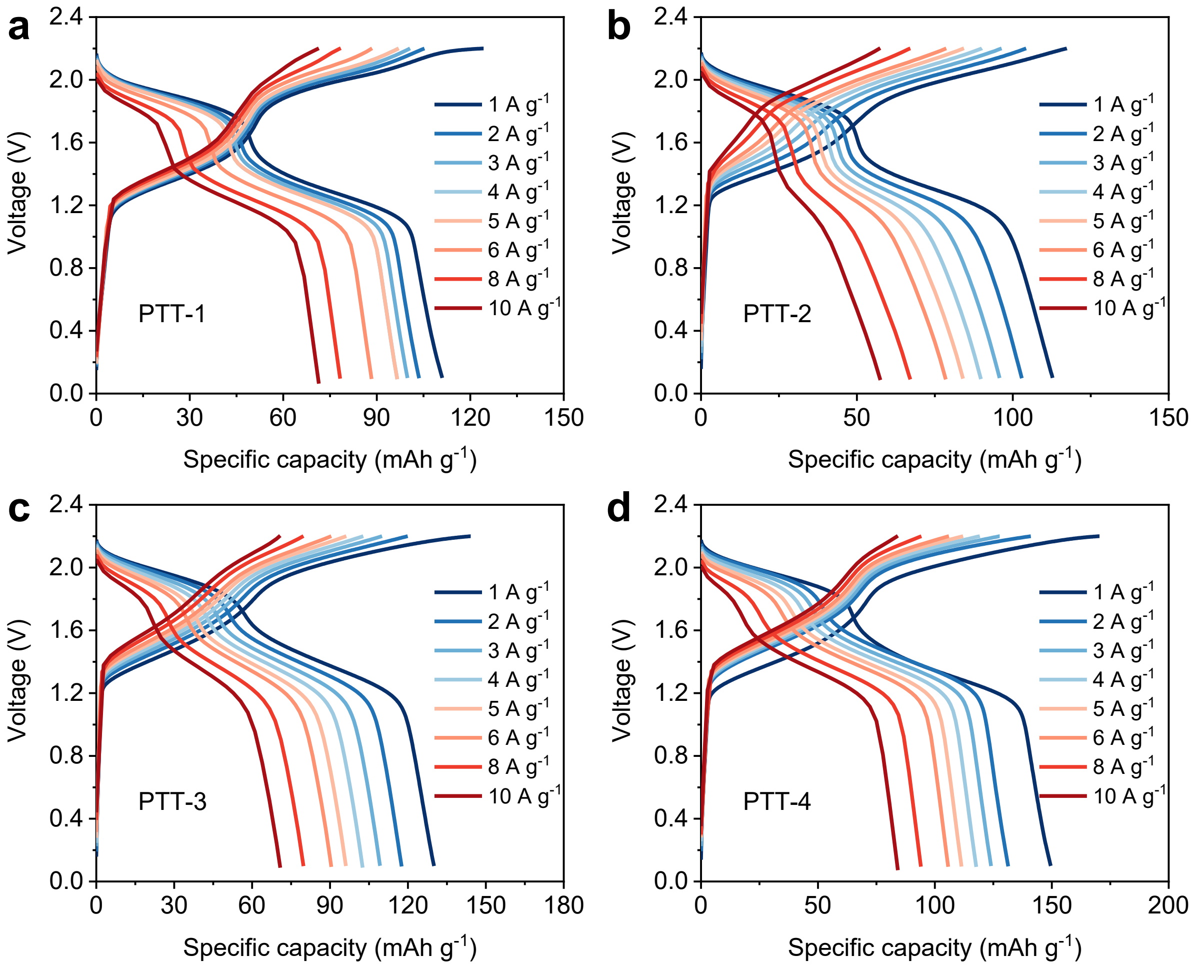

Figure S13. Rate performance of PTT-n (n=1, 2, 3, 4) at various current densities from 1 to 10 A g⁻¹. Voltage-capacity profiles of (a) PTT-1, (b) PTT-2, (c) PTT-3 and (d) PTT-4 cathodes.

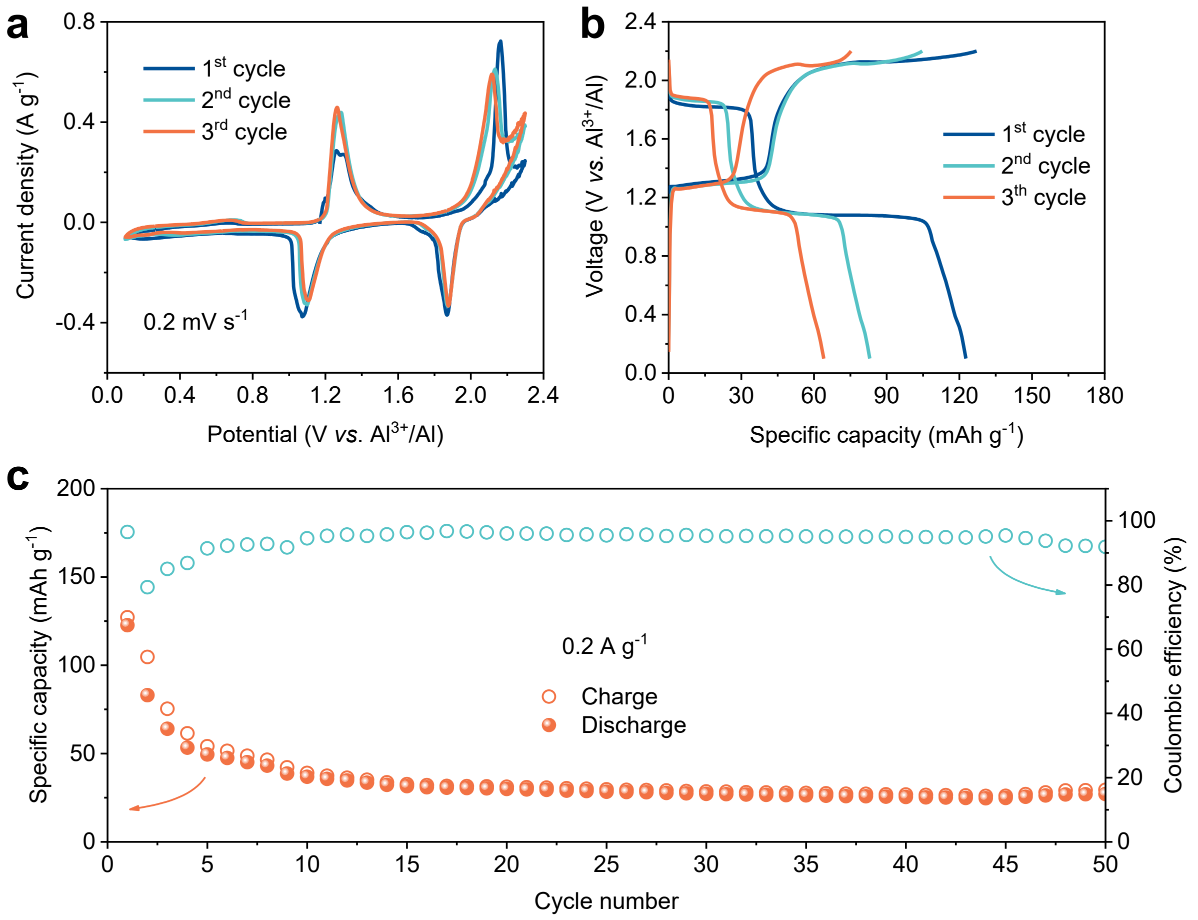

Figure S14. Electrochemical performance of TT cathode. (a) CV curves at 0.2 mV s^{-1}. (b) Galvanostatic discharge/charge curves at 0.2 A g^{-1}. (c) Cycling performance at 0.2 A g^{-1}.

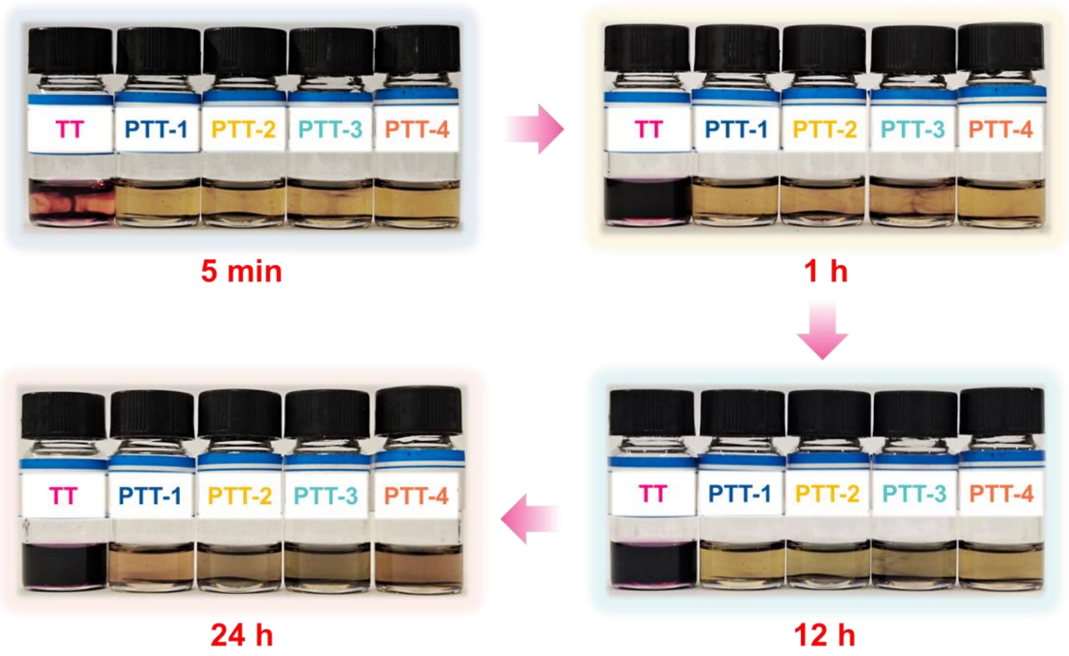

Figure S15. Solubility test of TT monomer and PTT-n (n=1, 2, 3, 4) polymers. Optical photographs of TT and PTT-n (n=1, 2, 3, 4) electrodes immersed in acidic ionic liquid electrolyte ($AlCl_3$/[EMIm]Cl=1.3) for different times.

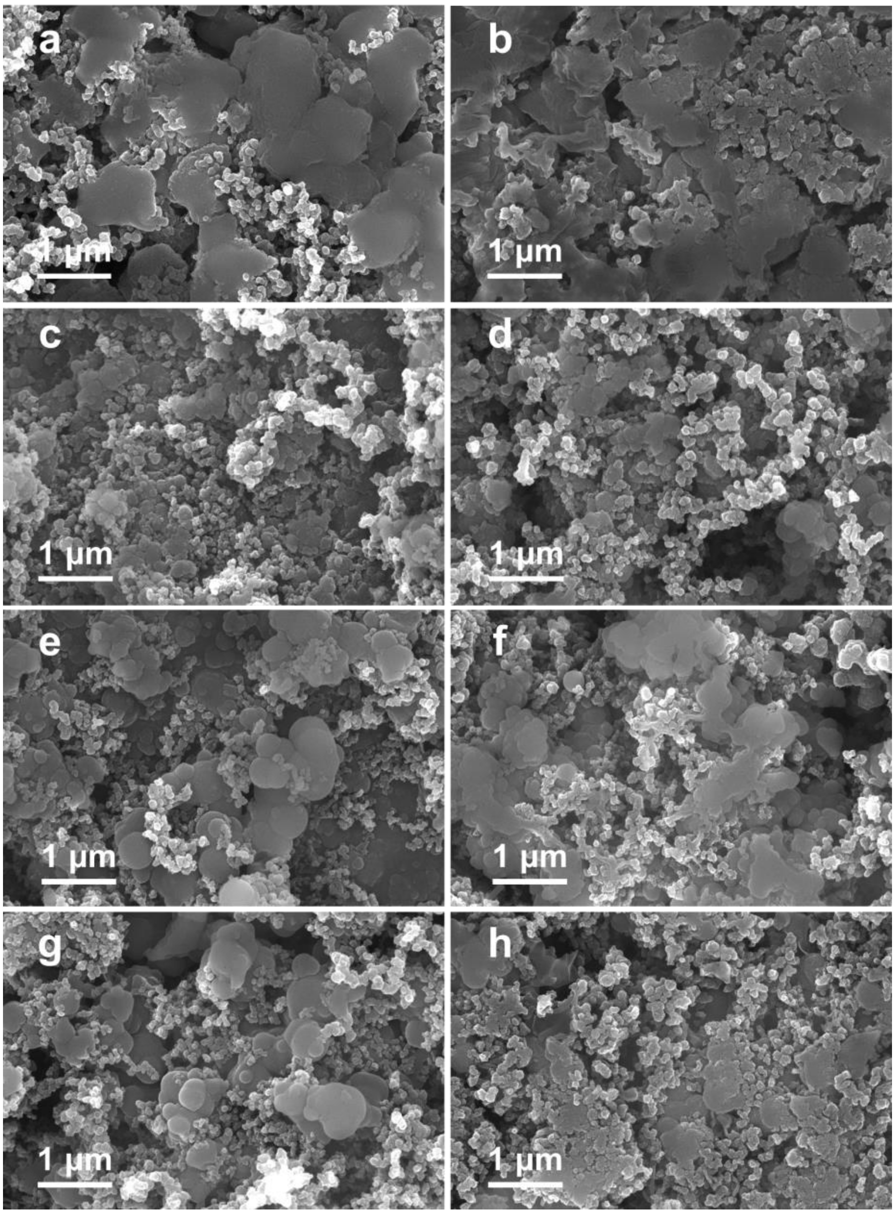

Figure S16. Morphology characterization of PTT-n (n=1, 2, 3, 4) electrodes. SEM images of (a,b) PTT-1, (c,d) PTT-2, (e,f) PTT-3, (g,h) PTT-4 electrodes before cycling and after 10,000 cycles.

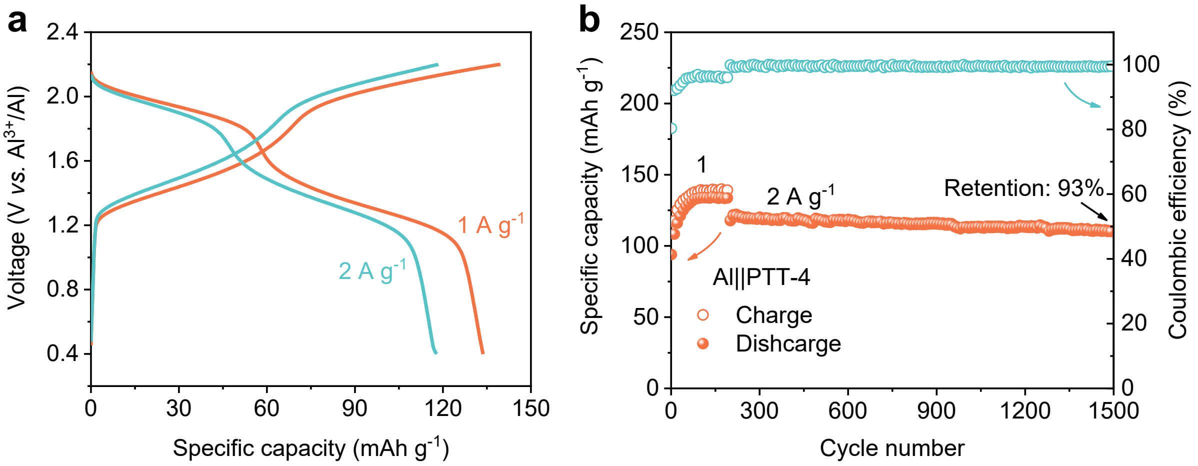

Figure S17. Electrochemical performance of the pouch-type Al||PTT-4 battery. (a) Galvanostatic discharge/charge curves and (b) cycling stability at 1 and 2 A g^{-1}.

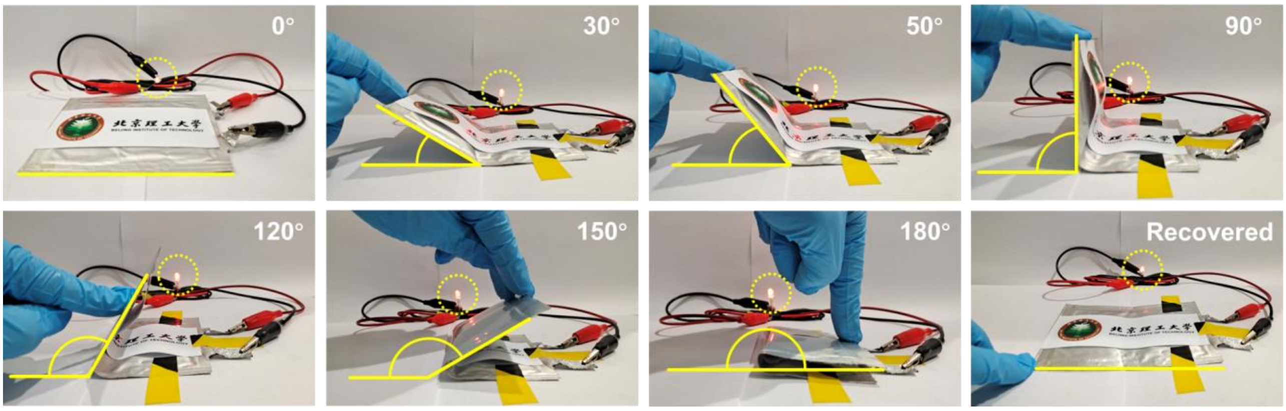

Figure S18. Bending test of the pouch-type Al||PTT-4 battery.

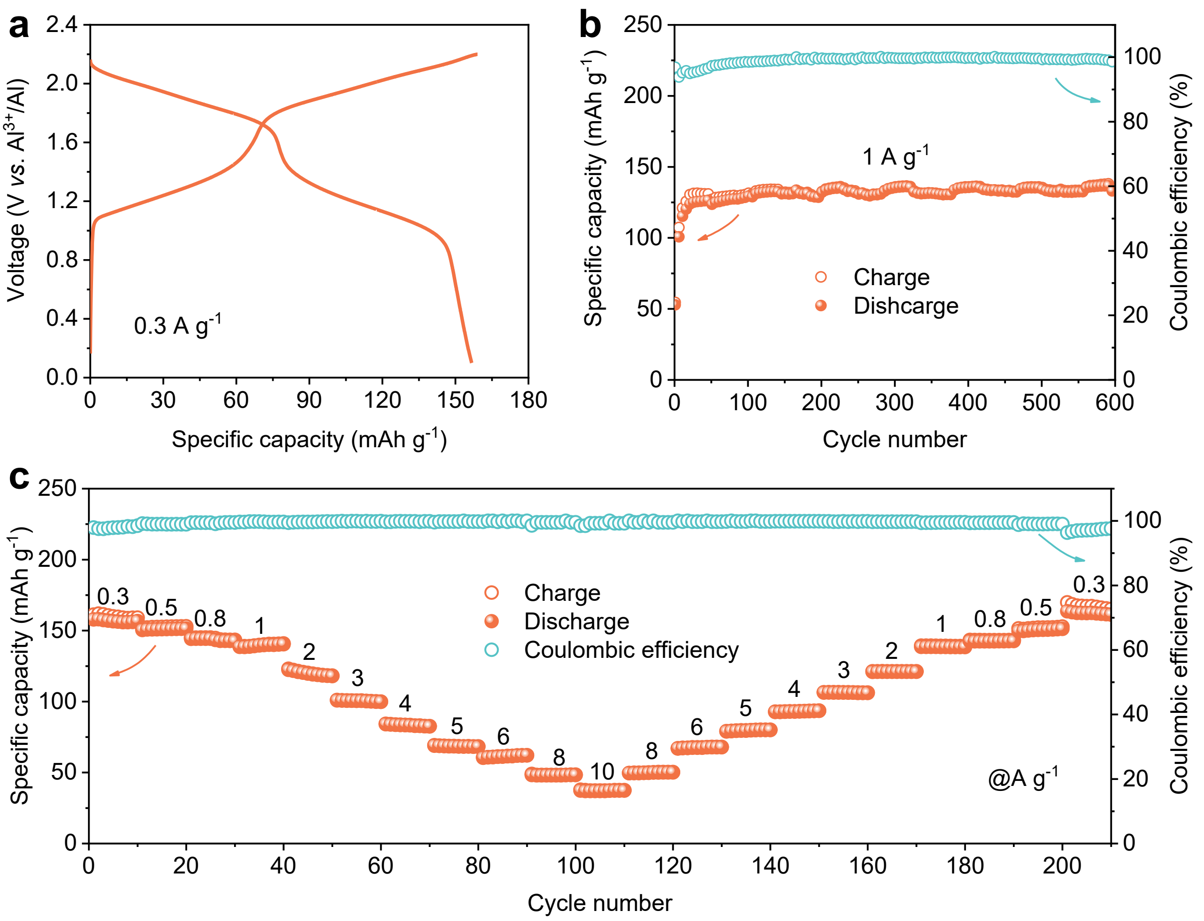

Figure S19. The electrochemical performance of PTT-4 cathode in $AlCl_3$/urea electrolyte (mole ratio: $AlCl_3$/urea = 1.3). (a) Galvanostatic discharge/charge curves at 0.3 A g^{-1}. (b) Cycling stability at 1 A g^{-1}. (c) Rate capability at various current densities from 0.3 to 10 A g^{-1}.

Note: The PTT-4 cathode delivers an average discharge voltage of 1.5 V and a capacity of 157 mAh g^{-1} at 0.3 A g^{-1}, corresponding to a high energy density of 236 Wh kg^{-1}.

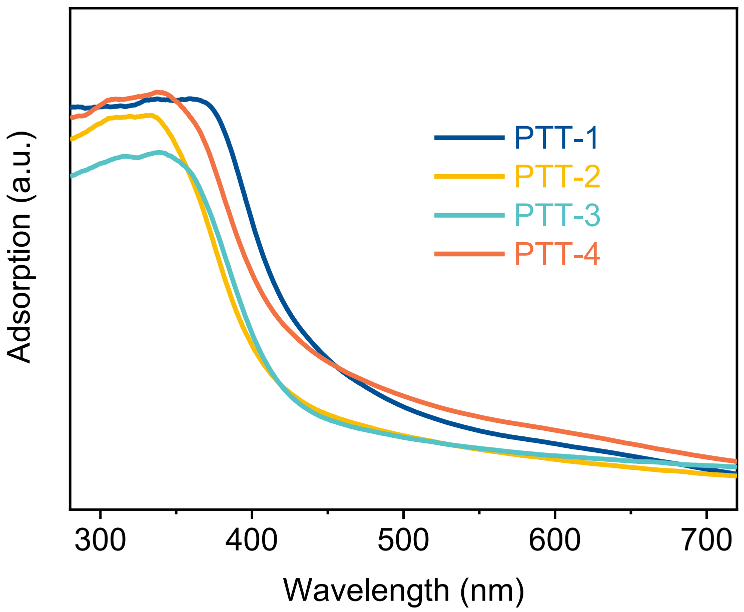

Figure S20. UV-vis absorption spectra of PTT-n (n=1, 2, 3, 4) polymers.

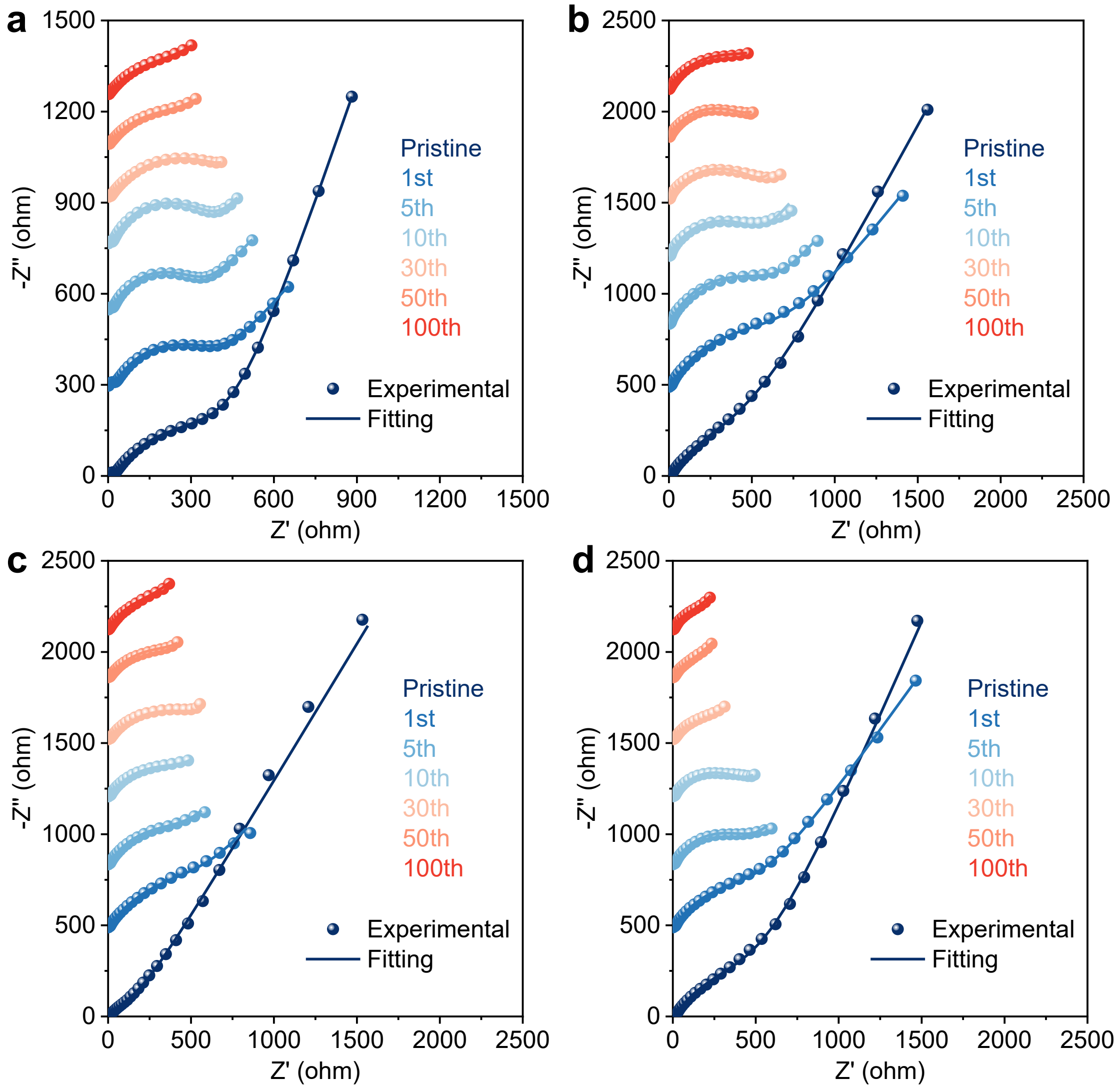

Figure S21. The electrochemical kinetics studies of PTT-n (n=1, 2, 3, 4) cathodes at different cycles. Electrochemical impedance spectroscopy (EIS) of (a) PTT-1, (b) PTT-2, (c) PTT-3 and (d) PTT-4 cathodes at different cycles from 0 to 100th cycle.

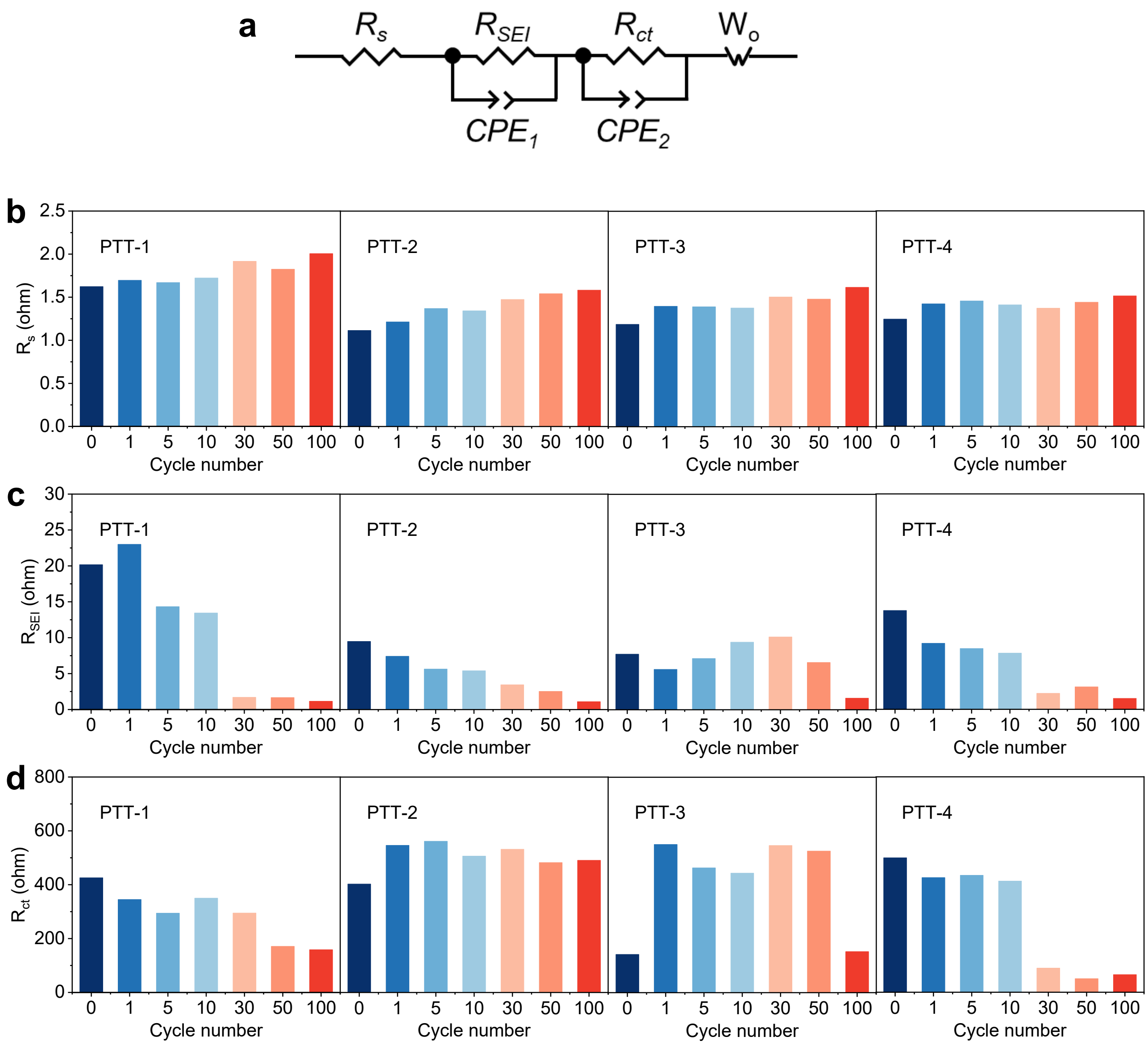

Figure S22. (a) The equivalent circuit diagram and fitting (b) R_s, (c) R_{SEI} and (d) R_{ct} values of PTT-n (n=1, 2, 3, 4) cathodes at different cycles. R_s, R_{SEI} and R_{ct} represent ohmic resistance, ion migration resistance in solid electrolyte interface (SEI) film and charge transfer resistance of electrochemical reaction, respectively.

Note: With the increase of cycle number, the R_s of PTT-n (n=1, 2, 3, 4) cathodes becomes stable, and the R_{SEI} and R_{ct} significantly decrease, indicating the continuously improved reaction kinetics.

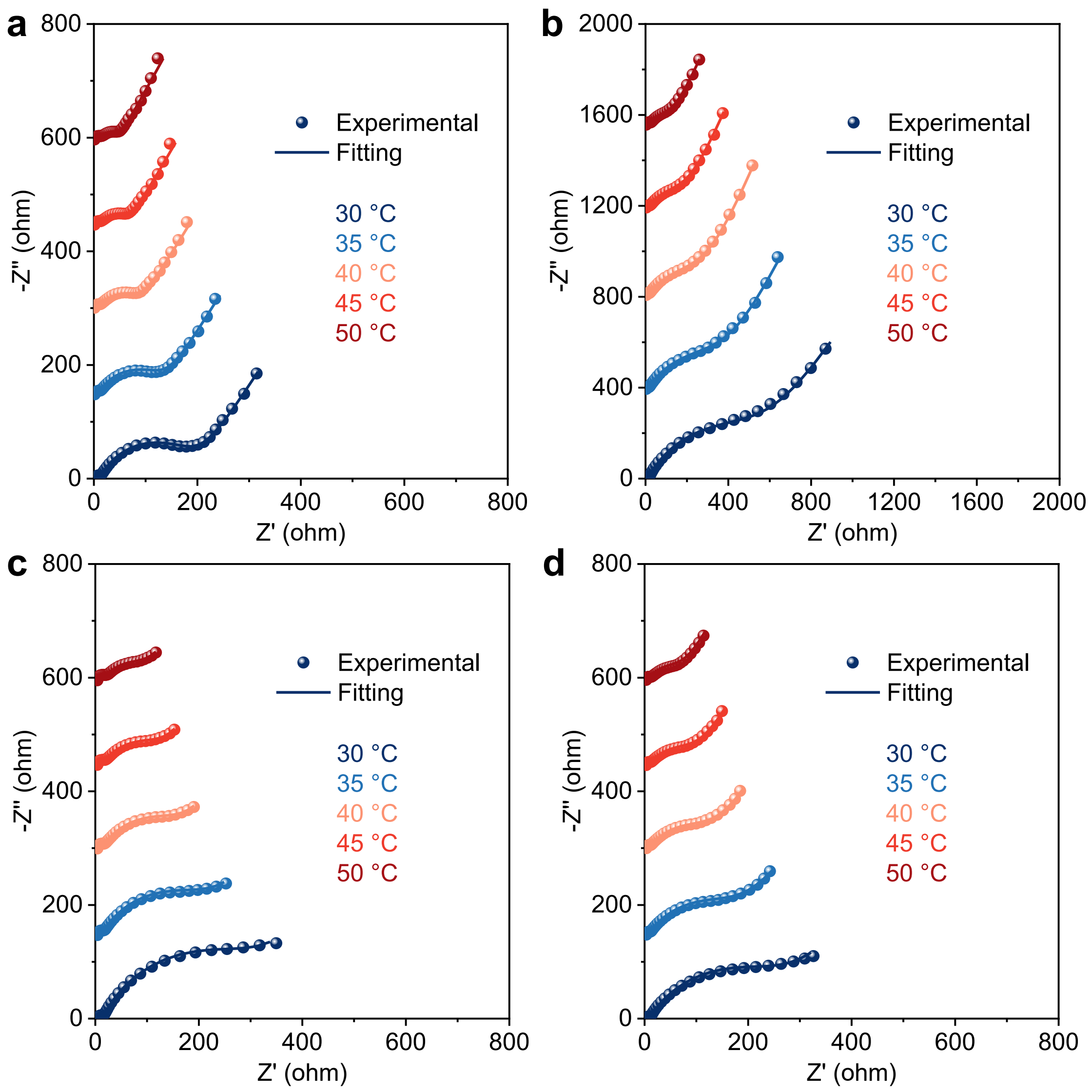

Figure S23. The electrochemical kinetics studies of PTT-n (n=1, 2, 3, 4) at different temperatures. Electrochemical impedance spectroscopy (EIS) of (a) PTT-1, (b) PTT-2, (c) PTT-3 and (d) PTT-4 cathodes at different temperatures from 30 to 50 °C.

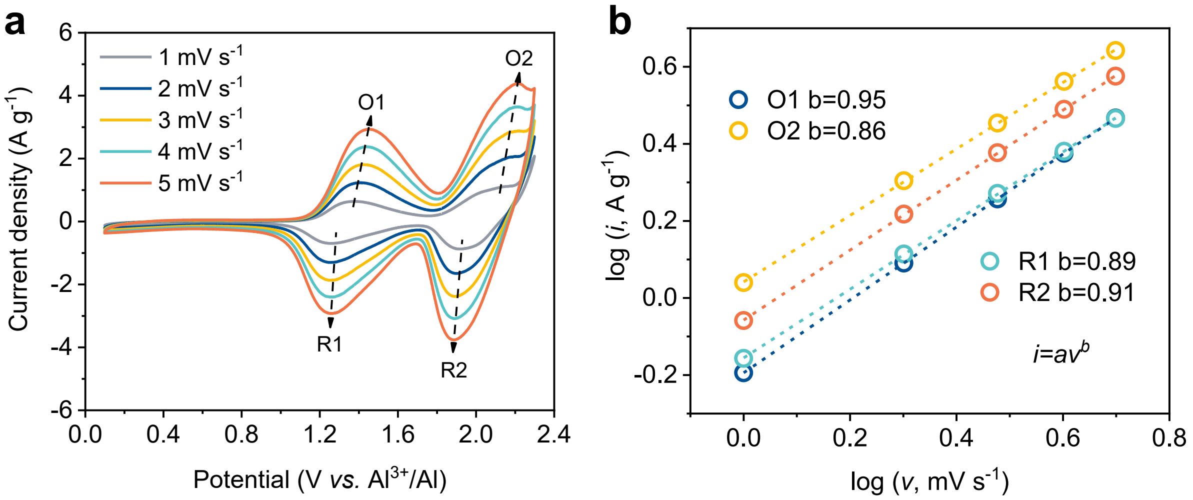

Figure S24. Analysis of Faradaic and non-faradaic processes of PTT-4 cathode. (a) CV curves at different scan rates from 1 to 5 mV s^{-1}. (b) Calculated b-values by linearly fitting the relationship of log(i) and log(v) (peak current: i, scan rate: v).

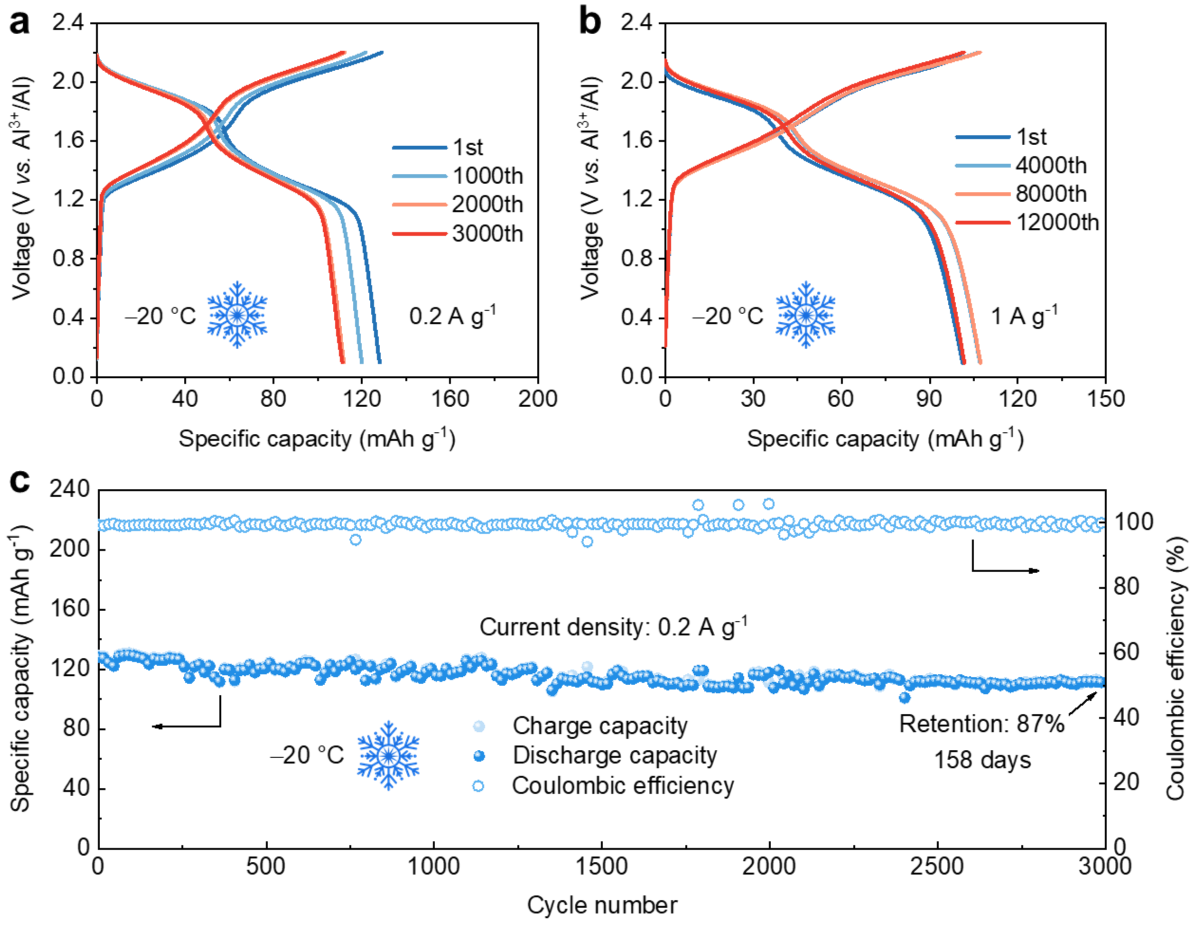

Figure S25. The low-temperature performance of PTT-4 cathode at −20 °C. Galvanostatic discharge/charge curves at (a) 0.2 A g^{-1} and (b) 1 A g^{-1}. (c) Long cycling stability at 0.2 A g^{-1}. The PTT-4 cathode delivers a capacity retention of ~87% even after cycling 158 days.

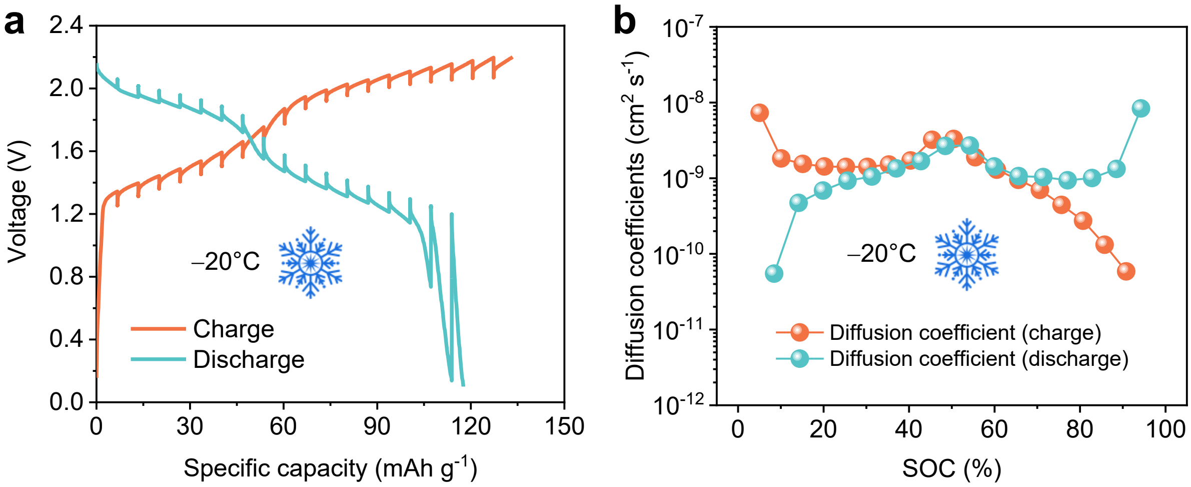

Figure S26. The ion diffusion kinetics studies of PTT-4 cathode at −20 °C. (a) GITT curves of PTT-4 cathode. (b) Calculated ion diffusion coefficient of Al-complex anions in PTT-4 cathode.

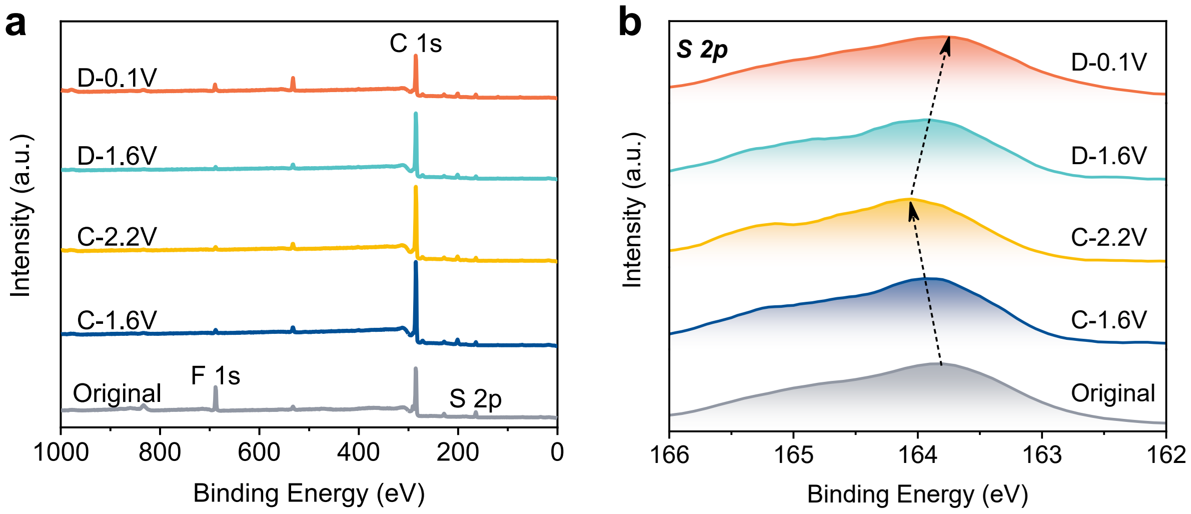

Figure S27. Analysis of charge storage mechanism for PTT-4. (a) Full survey XPS spectra and (b) S2p XPS spectra of PTT-4 cathode at different charge/discharge states.

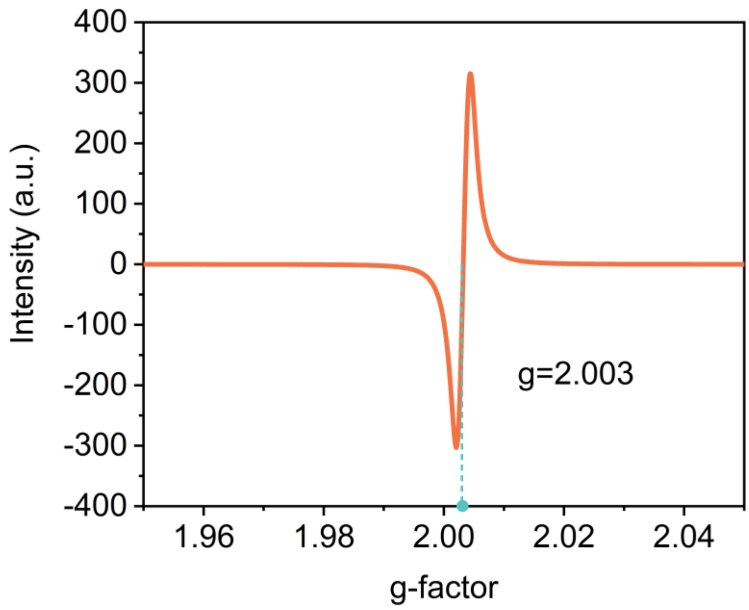

Figure S28. EPR spectrum of the PTT-4 cathode after charging to 2.2 V. The *g* value of C–S^{+}–C radicals is calculated to be 2.003.

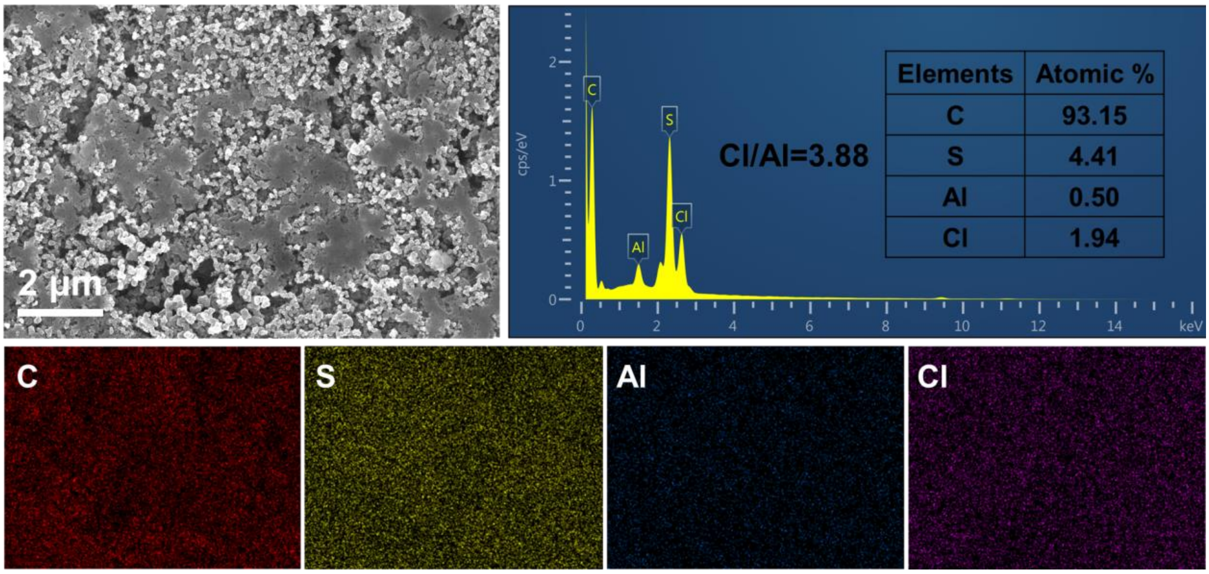

Elements	Atomic %
C	93.15
S	4.41
Al	0.50
Cl	1.94

Figure S29. EDS mapping of the PTT-4 cathode after fully charged to 2.2 V. SEM image and corresponding EDS mapping of C, S, Al and Cl.

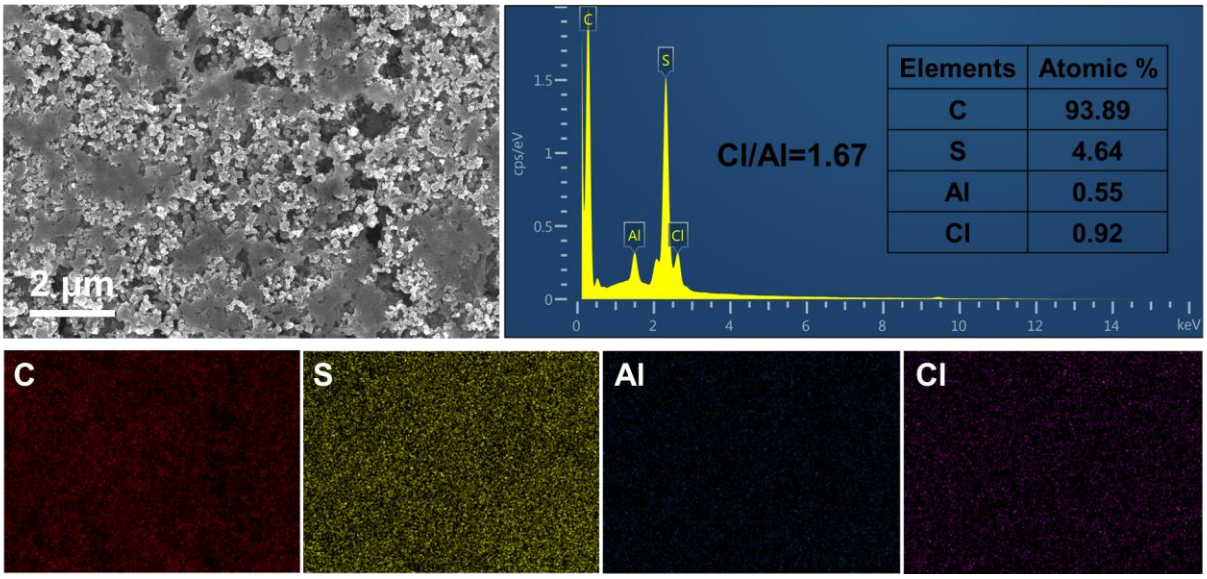

Figure S30. EDS mapping of the PTT-4 cathode after discharged to 0.1 V. SEM image and corresponding EDS mapping of C, S, Al and Cl.

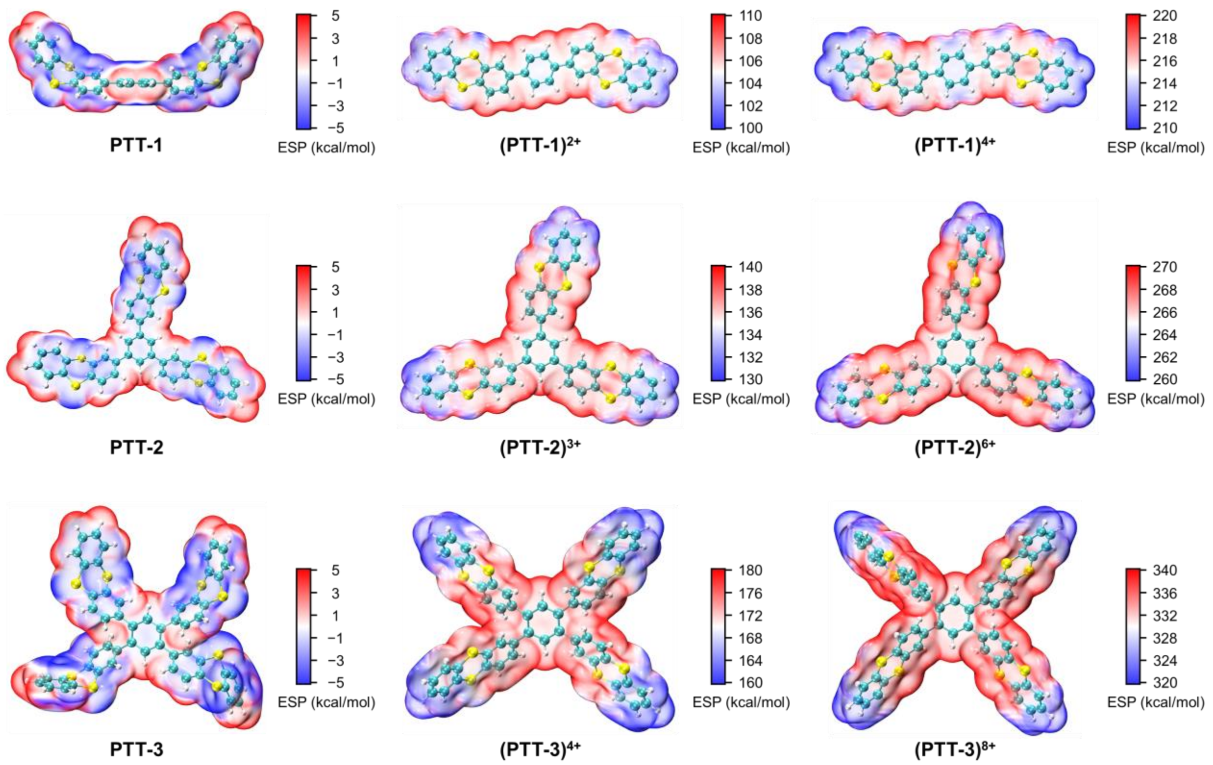

Figure S31. Molecular ESP distribution of PTT-1, PTT-2, PTT-3 and their oxidation-state species.

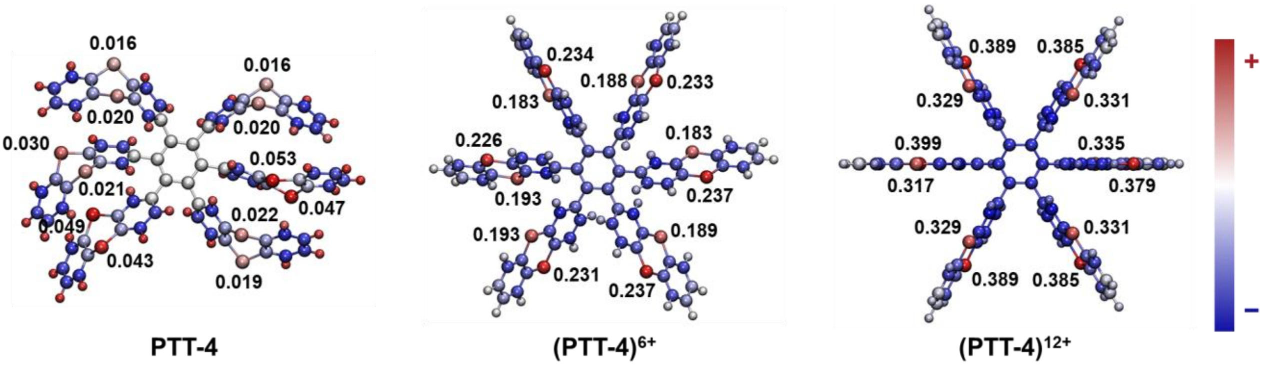

Figure S32. Atomic charge analysis of PTT-4, $(PTT\text{-}4)^{6+}$, and $(PTT\text{-}4)^{12+}$. Hirshfeld charges (HCs) of PTT-4, $(PTT\text{-}4)^{6+}$ and $(PTT\text{-}4)^{12+}$. The atoms colored in red and blue represent positive and negative HCs values, respectively.

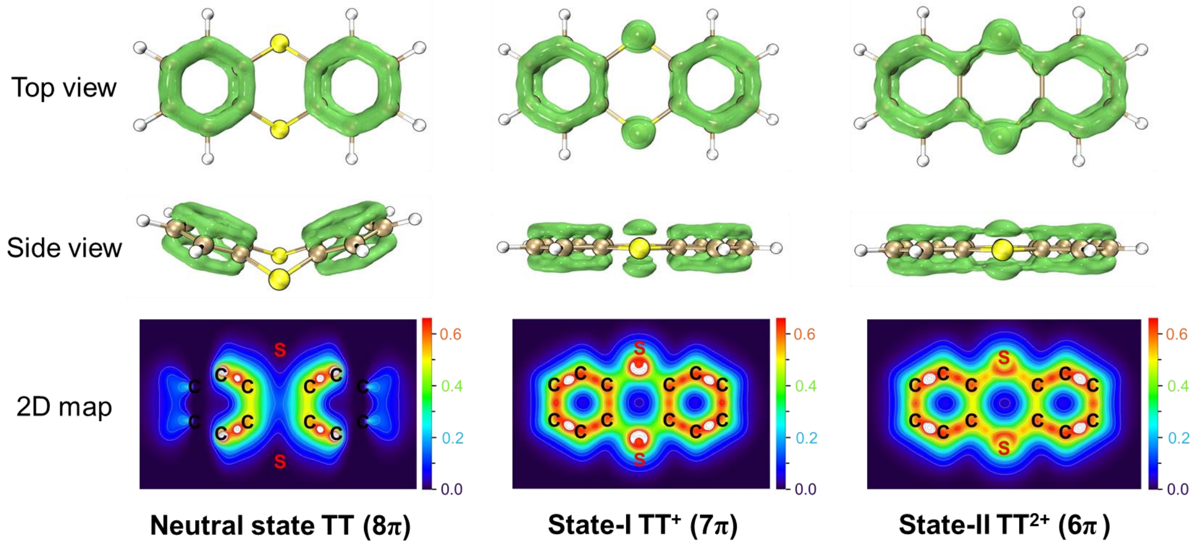

Figure S33. Electronic structure evolution of the TT unit during the oxidation process. LOL-π isosurface and 2D maps of the TT unit at different redox states.

Note: During the oxidation process, the sulfur heterocycle loses two π-electrons from 8π to 6π. The π-electron delocalization path of TT becomes more continuous, indicating an enhanced π-electron delocalization effect.

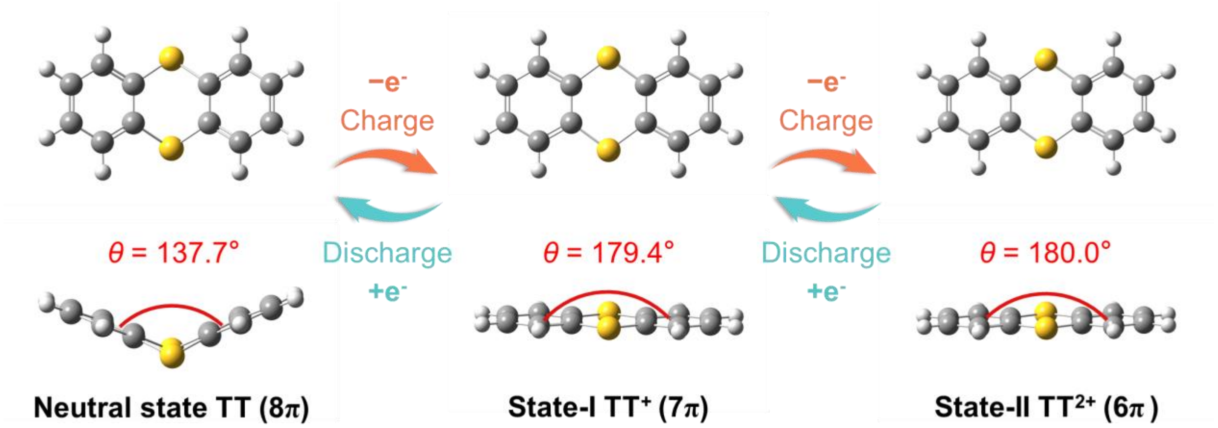

Figure S34. Molecular conformation evolution of the TT unit during the oxidation process.

Note: Due to the redox of sulfur heterocycle (oxidized from 8π to 6π/reduced from 6π to 8π), the TT unit undergoes a unique conformation conversion between non-planar and planar molecules.

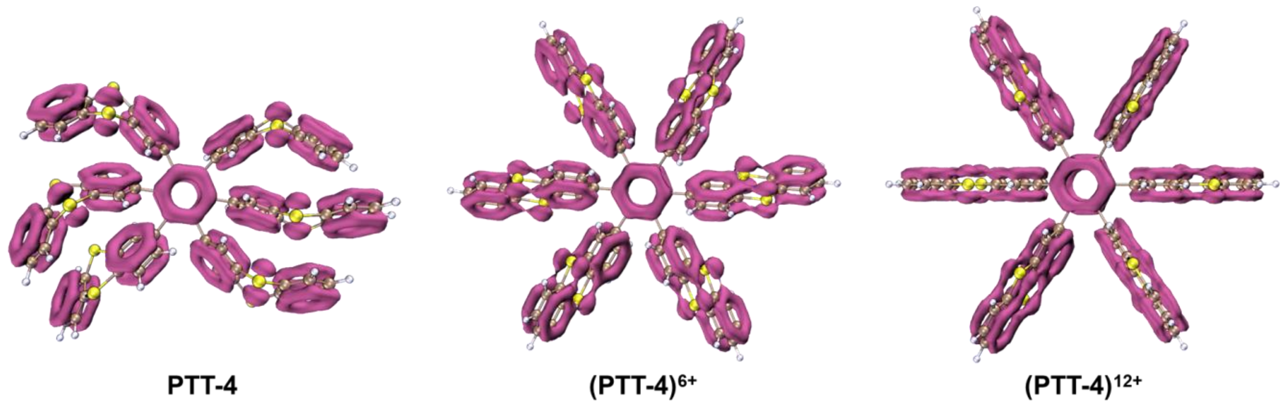

Figure S35. LOL-π isosurface maps of PTT-4 at different redox states.

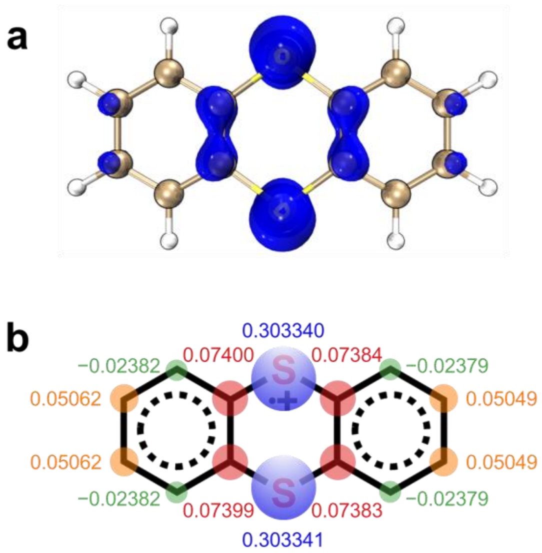

Figure S36. Electron spin density (ESD) analysis of radical intermediate TT^{+} (7π). (a) The simulated electron spin density and (b) calculated atomic spin population values of TT^{+} (7π).

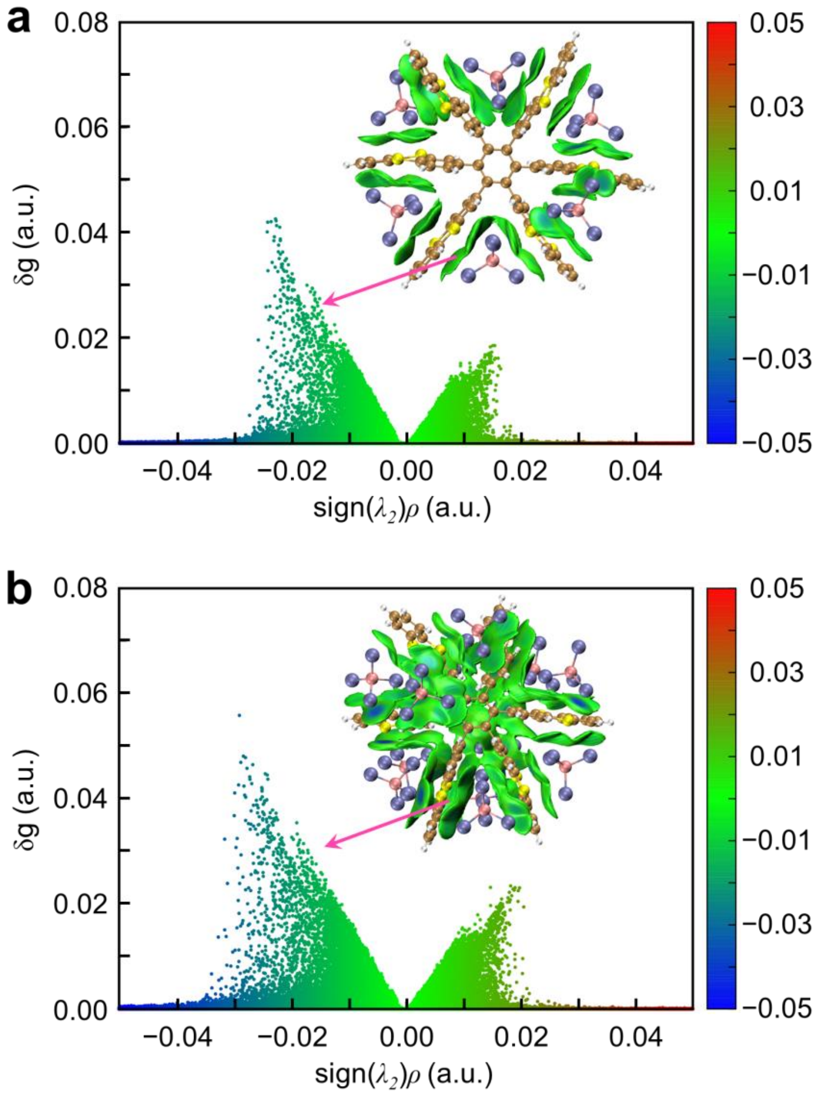

Figure S37. Weak interaction analysis of PTT-4 and $AlCl_4^-$ anions. Scatter plots and gradient isosurfaces of IGMH of (a) (PTT-4)-6$AlCl_4^-$ and (b) (PTT-4)-12$AlCl_4^-$.

Note: The detected green isosurfaces and corresponding spikes in the sign(λ_2)ρ from −0.02 to 0.00 a.u. indicate a weak interaction between TT unit and $AlCl_4^-$ anion in PTT-4.

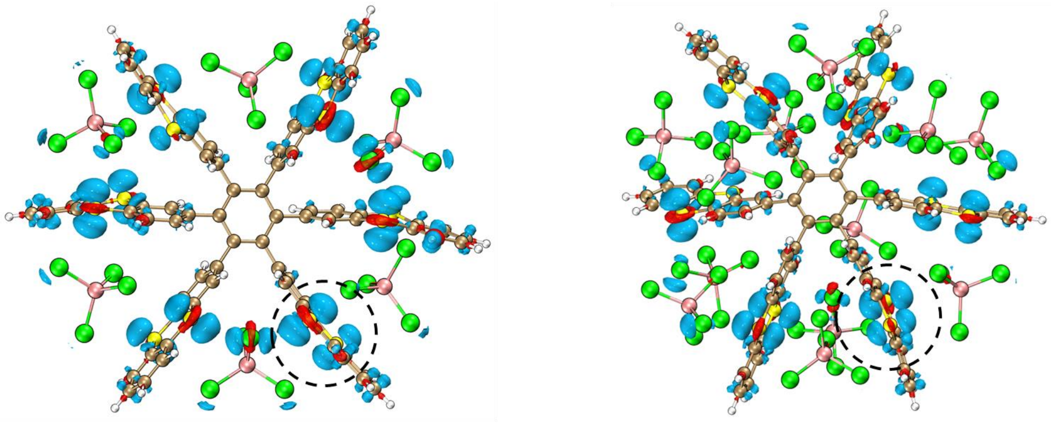

(PTT-4)-6$AlCl_4^-$ (PTT-4)-12$AlCl_4^-$

Figure S38. Electron density difference (EDD) plots of (PTT-4)-6$AlCl_4^-$ and (PTT-4)-12$AlCl_4^-$.

The blue and red isosurfaces denote the electron depletion and accumulation regions, respectively.

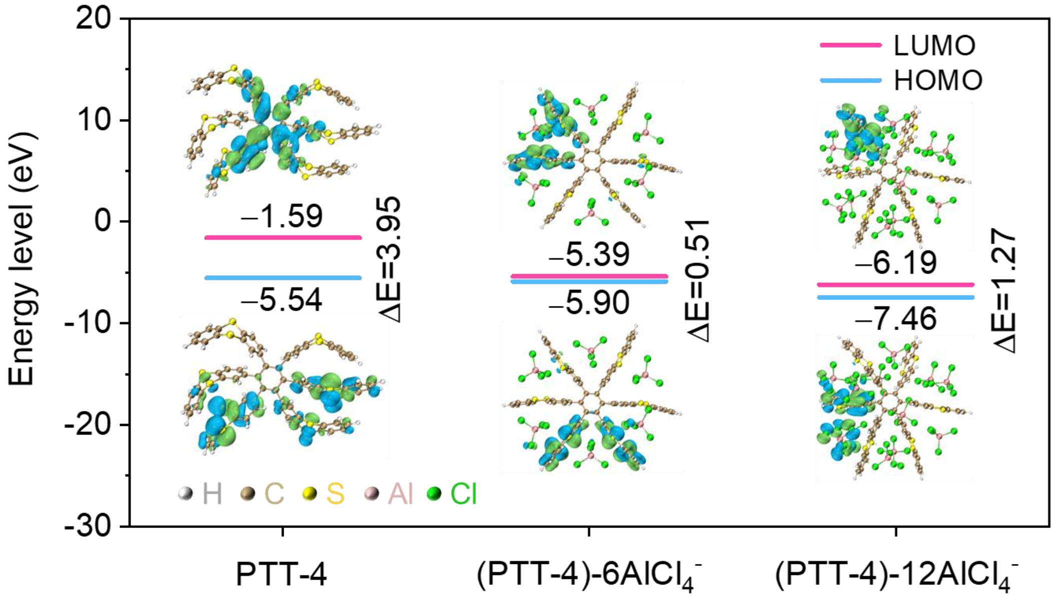

Figure S39. Calculated LUMO-HOMO energy levels of PTT-4, (PTT-4)-6$AlCl_4^-$, and (PTT-4)-12$AlCl_4^-$.

Table S1. Theoretical capacity calculation of PTT-n (n=1, 2, 3, 4) polymers based on each repeating structure unit for 2-electrons transfer.

Sulfur heterocyclic polymers	Molecular formula	Molecular weight per unit (g mol^{-1})	Theoretical capacity (mAh g^{-1})	Utilization rate of active sites (%)
PTT-1	$(C_{18}H_{10}S_2)_n$	290	184.8	60.0
PTT-2	$(C_{16}H_8S_2)_n$	264	203.0	54.7
PTT-3	$(C_{15}H_7S_2)_n$	251	213.6	60.9
PTT-4	$(C_{14}H_6S_2)_n$	238	225.2	66.6

Table S2. Comparison of average discharge voltage, specific capacity, energy density, cycling stability, rate capability, and mass loading of recently reported organic cathode materials (n-type: green, p-type: purple, bipolar-type: blue) and graphitic cathode materials for AIBs.

Organic and graphitic cathodes	Average discharge voltage (V)	Specific capacity (mAh g^{-1}@ A g^{-1})	Energy density (Wh kg^{-1})	Cycling stability (%@cycles @A g^{-1})	Rate capability (mAh g^{-1}@ A g^{-1})	Mass loading (mg@ cm^{-2})	Ref.
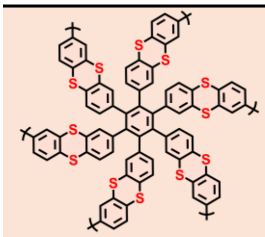 PTT-4	**1.7**	**150@1**	**255**	**90%@18000@10**	**110@10**	**1.0-1.5**	**This work**
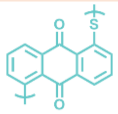 PAQS	1	190@0.125	176	60%@500@0.125	125@2.5	2.0	[12]
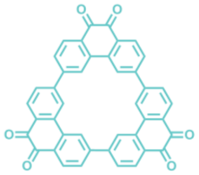 PQ-Δ	1.35	82@0.2	123	59%@5000@2	70@5	0.5	[13]
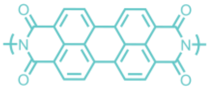 PI	1.25	83@1	104	88%@1800@1	40@10	1.0	[14]
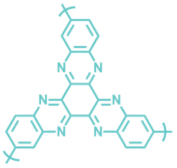 PHATN	0.7	145@0.05	102	63%@100@0.05	/	2.0	[15]
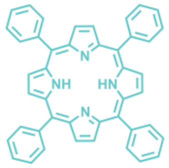 H_2TPP	1.2	101@0.1	126	96%@5000@0.2	74@0.5	1.1-1.3	[16]

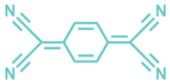 TCNQ	1.3	115@0.5	168	75%@2000@0.5	100@0.5	0.5	[17]
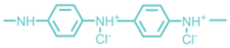 PANI(H^+)	0.95	200@1	190	88%@8000@10	75@40	1.1	[18]
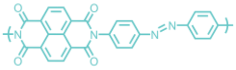 PNA	1.1	178@0.05	231	97%@10000@2	51@5	1.0	[19]
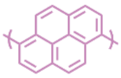 Polypyrene	1.7	70@0.2	119	45%@300@0.2	/	0.5	[20]
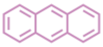 Anthracene	1.3	157@0.1	236	83%@800@0.1	82@0.5	1.0	[21]
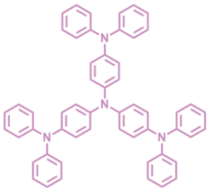 Amine	1.1	135@0.1	148.5	94%@4000@1	116@2	3.5-4.0	[22]
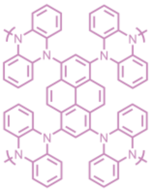 PyPz	0.83	231@0.1	192	78%@100000 @10	170@10	1.4	[23]
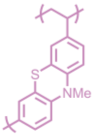 X-PVMPT	1.1	167@0.11	184	88%@5000@2.2	65@22	1.2-1.7	[24]
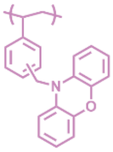 PVBPX	1.4	133@0.2	186	97%@50000@5	60@10	1.0	[25]
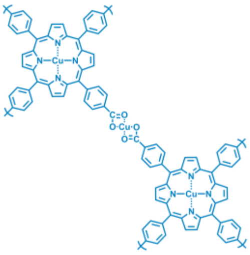 2D Cu-MOF	0.96	184@0.05	177	94%@1000@0.2	65@0.5	1.0	[26]

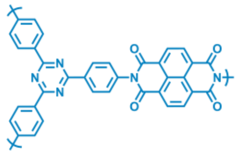 2D NT-COF	1.3	132@0.1	172	97%@4000@1	80@1.6	1.0-2.0	[27]
Graphite paper	1.8	85@0.05	153	100%@50@0.05	63@150	/	[28]
Pyrolytic graphite	1.9	66@0.066	125	100%@200 @0.264	25@0.264	1.0-5.0	[29]
3D Graphitic foam	1.9	60@4	114	100%@7500@4	60@5	1.0-5.0	[29]
Monolithic 3D graphitic foam	1.8	60@2	108	100%@4000@12	60@12	1.0	[30]
Natural graphite	1.9	110@0.066	209	100%@6000 @0.66	60@0.66	3.0-4.0	[31]

Table S3. Elemental analysis of PTT-n (n=1,2,3,4) polymers.

Samples	C (%)	H (%)	S (%)	Br (%)	B (%)	O (%)
PTT-1 $(C_{18}H_{10}S_2)_nBrBO_2C_6H_{12}$	70.078	3.192	25.222	0.981	0.135	0.392
PTT-2 $(C_{16}H_8S_2)_nBrBO_2C_6H_{12}$	68.155	3.613	15.982	7.967	1.096	3.187
PTT-3 $(C_{15}H_7S_2)_nBrBO_2C_6H_{12}$	68.827	3.366	19.063	5.687	0.782	2.275
PTT-4 $(C_{14}H_6S_2)_nBrBO_2C_6H_{12}$	66.087	3.308	24.172	4.184	0.575	1.674

Note: The small amounts of Br, B, O and elements could be ascribed to the end groups. The weight ratio of Br, B and O was obtained by calculation.

Table S4. The fitting resistance values (R_s, R_{SEI} and R_{ct}) of PTT-n (n=1,2,3,4) cathodes at different cycles.

		Pristine	1st	5th	10th	30th	50th	100th
**PTT-1**	R_s	1.623	1.696	1.67	1.723	1.917	1.825	2.006
	R_{SEI}	20.17	23	14.32	13.44	1.694	1.666	1.144
	R_{ct}	425.9	345.1	294.4	350.1	294.8	171.1	158.6
**PTT-2**	R_s	1.115	1.215	1.37	1.343	1.475	1.542	1.583
	R_{SEI}	9.491	7.424	5.651	5.413	3.457	2.535	1.103
	R_{ct}	403	546.6	561.7	506.5	532.1	482.5	491
**PTT-3**	R_s	1.188	1.398	1.392	1.378	1.506	1.481	1.618
	R_{SEI}	7.75	5.623	7.139	9.416	10.15	6.581	1.615
	R_{ct}	142	550.5	463.6	443.8	546.6	526	152.6
**PTT-4**	R_s	1.244	1.421	1.455	1.41	1.371	1.44	1.514
	R_{SEI}	13.77	9.193	8.478	7.827	2.228	3.141	1.531
	R_{ct}	499.6	425.7	434.5	413	89.84	50.68	65.54

Table S5. The calculated Gibbs free energies (G) of PTT-4 and its complexes (PTT-4)-$n$$AlCl_4^-$ (n=3, 6, 9, 12), as well as the Gibbs free energy differences (ΔG) of the discharge reaction processes.

Compounds	G (hartree)	Discharge reaction process	ΔG (eV)
PTT-4	−7776.942	/	/
$AlCl_4^-$	−2083.809	/	/
(PTT-4)-12$AlCl_4^-$	−32780.817	(PTT-4)-12$AlCl_4^-$ + 3e^- → (PTT-4)-9$AlCl_4^-$ + 3$AlCl_4^-$	$\Delta G_1 = -13.92$
(PTT-4)-9$AlCl_4^-$	−26529.901	(PTT-4)-9$AlCl_4^-$ + 3e^- → (PTT-4)-6$AlCl_4^-$ + 3$AlCl_4^-$	$\Delta G_2 = -11.32$
(PTT-4)-6$AlCl_4^-$	−20278.890	(PTT-4)-6$AlCl_4^-$ + 3e^- → (PTT-4)-3$AlCl_4^-$ + 3$AlCl_4^-$	$\Delta G_3 = -12.48$
(PTT-4)-3$AlCl_4^-$	−14027.921	(PTT-4)-3$AlCl_4^-$ + 3e^- → PTT-4 + 3$AlCl_4^-$	$\Delta G_4 = -12.22$

REFERENCES

1. Mohamed M G, Chaganti S V, Li M-S *et al*. Ultrastable porous organic polymers containing thianthrene and pyrene units as organic electrode materials for supercapacitors. *ACS Appl Energy Mater* 2022; **5**: 6442-52.
2. Luo L-W, Ma W, Dong P *et al*. Synthetic control of electronic property and porosity in anthraquinone-based conjugated polymer cathodes for high-rate and long-cycle-life Na–organic batteries. *ACS Nano* 2022; **16**: 14590-9.
3. Frisch M J, Trucks G W, Schlegel H B *et al*. Gaussian 16 Rev. C.01. 2016.
4. Stephens P J, Devlin F J, Chabalowski C F *et al*. Ab initio calculation of vibrational absorption and circular dichroism spectra using density functional force fields. *J Phys Chem* 1994; **98**: 11623-7.
5. Weigend F&Ahlrichs R. Balanced basis sets of split valence, triple zeta valence and quadruple zeta valence quality for H to Rn: design and assessment of accuracy. *Phys Chem Chem Phys* 2005; **7**: 3297-305.
6. Lu T&Chen Q. Shermo: A general code for calculating molecular thermochemistry properties. *Comput Theor Chem* 2021; **1200**: 113249.
7. Alecu I M, Zheng J, Zhao Y *et al*. Computational thermochemistry: scale factor databases and scale factors for vibrational frequencies obtained from electronic model chemistries. *J Chem Theory Comput* 2010; **6**: 2872-87.
8. Lu T&Chen F. Multiwfn: A multifunctional wavefunction analyzer. *J Comput Chem* 2012; **33**: 580-92.
9. Humphrey W, Dalke A&Schulten K. VMD: visual molecular dynamics. *J Mol Graphics* 1996; **14**: 33-8.
10. Geuenich D, Hess K, Köhler F *et al*. Anisotropy of the induced current density (ACID), a general method to quantify and visualize electronic delocalization. *Chem Rev* 2005; **105**: 3758-72.
11. Song Z, Miao L, Duan H *et al*. Anionic co-insertion charge storage in dinitrobenzene cathodes for high-performance aqueous zinc–organic batteries. *Angew Chem Int Ed* 2022; **61**: e202208821.
12. Bitenc J, Lindahl N, Vizintin A *et al*. Concept and electrochemical mechanism of an Al metal

anode–organic cathode battery. *Energy Stor Mater* 2020; **24**: 379-83.

13. Kim D J, Yoo D J, Otley M T *et al*. Rechargeable aluminium organic batteries. *Nat Energy* 2019; **4**: 51-9.
14. Zhou J, Yu X, Zhou J *et al*. Polyimide/metal-organic framework hybrid for high performance Al–organic battery. *Energy Stor Mater* 2020; **31**: 58-63.
15. Mao M, Luo C, Pollard T P *et al*. A pyrazine-based polymer for fast-charge batteries. *Angew Chem Int Ed* 2019; **58**: 17820-6.
16. Han X, Li S, Song W-L *et al*. Stable high-capacity organic aluminum-porphyrin batteries. *Adv Energy Mater* 2021; **11**: 2101446.
17. Guo F, Huang Z, Wang M *et al*. Active cyano groups to coordinate $AlCl_2^+$ cation for rechargeable aluminum batteries. *Energy Stor Mater* 2020; **33**: 250-7.
18. Wang S, Huang S, Yao M *et al*. Engineering active sites of polyaniline for $AlCl_2^+$ storage in an aluminum-ion battery. *Angew Chem Int Ed* 2020; **59**: 11800-7.
19. Qin K, Tan S, Mohammadiroudbari M *et al*. Synergy of carbonyl and azo chemistries for wide-temperature-range rechargeable aluminum organic batteries. *Nano Energy* 2022; **101**: 107554.
20. Walter M, Kravchyk K V, Böfer C *et al*. Polypyrenes as high-performance cathode materials for aluminum batteries. *Adv Mater* 2018; **30**: 1705644.
21. Kong D, Cai T, Fan H *et al*. Polycyclic aromatic hydrocarbons as a new class of promising cathode materials for aluminum-ion batteries. *Angew Chem Int Ed* 2022; **61**: e202114681.
22. Wang G, Dmitrieva E, Kohn B *et al*. An efficient rechargeable aluminium–amine battery working under quaternization chemistry. *Angew Chem Int Ed* 2022; **61**: e202116194.
23. Ma W, Luo L-W, Huang X *et al*. Dihydrophenazine-based conjugated microporous polymer cathodes with enhanced electronic and ionic conductivities for high-performance aluminum dual-ion batteries. *Adv Energy Mater* 2023; **13**: 2203253.
24. Studer G, Schmidt A, Büttner J *et al*. On a high-capacity aluminium battery with a two-electron phenothiazine redox polymer as a positive electrode. *Energy Environ Sci* 2023; **16**: 3760-9.
25. Yang Z, Huang X, Meng P *et al*. Phenoxazine polymer-based p-type positive electrode for aluminum-ion batteries with ultra-long cycle life. *Angew Chem Int Ed* 2023; **62**: e202216797.

26. Guo Y, Wang W, Lei H *et al*. Alternate storage of opposite charges in multisites for high-energy-density Al–MOF batteries. *Adv Mater* 2022; **34**: 2110109.
27. Liu Y, Lu Y, Hossain Khan A *et al*. Redox-bipolar polyimide two-dimensional covalent organic framework cathodes for durable aluminium batteries. *Angew Chem Int Ed* 2023; **62**: e202306091.
28. Sun H, Wang W, Yu Z *et al*. A new aluminium-ion battery with high voltage, high safety and low cost. *Chem Commun* 2015; **51**: 11892-5.
29. Lin M, Gong M, Lu B *et al*. An ultrafast rechargeable aluminium-ion battery. *Nature* 2015; **520**: 324-8.
30. Wu Y, Gong M, Lin M-C *et al*. 3D Graphitic foams derived from chloroaluminate anion intercalation for ultrafast aluminum-ion battery. *Adv Mater* 2016; **28**: 9218-22.
31. Wang D, Wei C, Lin M *et al*. Advanced rechargeable aluminium ion battery with a high-quality natural graphite cathode. *Nat Commun* 2017; **8**: 14283.
