# Supplementary material for: A multisite super-crosslinked sulfur-heterocyclic polymer cathode for high-voltage and low-temperature aluminum–organic batteries
Source: Natl Sci Rev. 2025 Nov 22;13(1):nwaf526. doi: 10.1093/nsr/nwaf526 (PMC12796798; doi:10.1093/nsr/nwaf526)
Supplement: nwaf526_Supplemental_Files [file nwaf526_supplemental_files.zip › Teaser text.docx]

This work designs a multisite, super-crosslinked sulfur-heterocyclic polymer cathode with weak electron-donating effect and multi-electron transfer capability, thus achieving high operating voltage and superior low-temperature durability in aluminum-organic batteries.
